# RAC1 controls progressive movement and competitiveness of mammalian spermatozoa

**DOI:** 10.1371/journal.pgen.1009308

**Published:** 2021-02-04

**Authors:** Alexandra Amaral, Bernhard G. Herrmann

**Affiliations:** 1 Department of Developmental Genetics, Max Planck Institute for Molecular Genetics, Berlin, Germany; 2 Institute for Medical Genetics, Charité–University Medicine Berlin, Campus Benjamin-Franklin, Berlin, Germany; The University of Melbourne, AUSTRALIA

## Abstract

Mammalian spermatozoa employ calcium (Ca^2+^) and cyclic adenosine monophosphate (cAMP) signaling in generating flagellar beat. However, how sperm direct their movement towards the egg cells has remained elusive. Here we show that the Rho small G protein RAC1 plays an important role in controlling progressive motility, in particular average path velocity and linearity. Upon RAC1 inhibition of wild type sperm with the drug NSC23766, progressive movement is impaired. Moreover, sperm from mice homozygous for the genetically variant *t*-haplotype region (*t*^*w5*^*/t*^*w32*^), which are sterile, show strongly enhanced RAC1 activity in comparison to wild type (+/+) controls, and quickly become immotile *in vitro*. Sperm from heterozygous (*t/+*) males, on the other hand, display intermediate RAC1 activity, impaired progressive motility and transmission ratio distortion (TRD) in favor of *t*-sperm. We show that *t*/+-derived sperm consist of two subpopulations, highly progressive and less progressive. The majority of highly progressive sperm carry the *t*-haplotype, while most less progressive sperm contain the wild type (+) chromosome. Dosage-controlled RAC1 inhibition in *t*/+ sperm by NSC23766 rescues progressive movement of (+)-sperm *in vitro*, directly demonstrating that impairment of progressive motility in the latter is caused by enhanced RAC1 activity. The combined data show that RAC1 plays a pivotal role in controlling progressive motility in sperm, and that inappropriate, enhanced or reduced RAC1 activity interferes with sperm progressive movement. Differential RAC1 activity within a sperm population impairs the competitiveness of sperm cells expressing suboptimal RAC1 activity and thus their fertilization success, as demonstrated by *t*/+-derived sperm. In conjunction with *t*-haplotype triggered TRD, we propose that Rho GTPase signaling is essential for directing sperm towards the egg cells.

## Author summary

Spermatozoa are highly specialized cells designed for one particular purpose: to swim towards the egg cells and fertilize them. Swimming is achieved by propelling rotation of the flagellum, a complex molecular motor. Many proteins building the flagellum have been identified, and mechanisms generating forward movement have been elucidated in some detail. However, how movement is directed towards the egg cells, is not well understood. In this study, we provide evidence that the Rho small GTPase RAC1 plays a pivotal role in sperm progressive movement. We show that inappropriately high or low RAC1 activity impairs progressive movement and the competitiveness of sperm within a sperm population. Sperm with balanced RAC1 activity therefore gain a selective advantage in reaching and fertilizing egg cells, as exemplified by the mouse *t*-haplotype. Our data highlight the importance of Rho signaling in controlling progressive motility, in sperm competition, non-Mendelian inheritance and male (in)fertility.

## Introduction

Forward movement requires two principal components, a driving force and steering. Mammalian sperm motility is complex. Flagellar movement is driven by the phosphorylation of axonemal outer dynein arms followed by ATP hydrolysis, which generates force effecting sliding of adjacent outer microtubule doublets in the axoneme and flagellar bending (for reviews see [1,2]). Several upstream regulatory events are involved, of which calcium (Ca^2+^) signaling and cyclic adenosine monophosphate (cAMP)-dependent protein kinase A (PKA) pathways are central. CatSper, the main known Ca^2+^ channel in the mammalian sperm flagellar membrane, is controlled by voltage and pHi, which are in turn controlled by potassium (K^+^) and sodium (Na^+^) channels, and by ligands from the oviductal fluid [3]. A rise in the internal Ca^2+^ levels, together with an increase in the concentration of bicarbonate (HCO_3_^-^) prompted by specific cellular transporters activate the atypical soluble adenylyl cyclase (sAC), which generates cAMP. This leads to PKA activation and ultimately results in a cascade of protein phosphorylation events, elicited by sperm tyrosine kinases and balanced by serine/tyrosine phosphatases, together regulating motility [4]. The putative protein targets here are numerous and any of them may influence the way sperm move.

Progressive movement is mainly controlled *via* outer dynein arms (ODA), while inner dynein arms (IDA) control the formation and propagation of flagellar bending [5–8]. However, how ODA and IDA activity are coordinated enabling directed movement of sperm towards the egg cell(s) remains elusive.

During fertilization, sperm are competing against each other for reaching the egg cells first. Though Mendelian genetics predicts that all sperm involved in the “fertilization race” have an equal chance of success, there is one exemplary case in mammals known to break Mendel’s rules, the well-studied *t*-haplotype in mouse. The *t*-haplotype is a genetic variant region of some 40 Mb on chromosome 17 encoding several factors causing transmission ratio distortion (TRD) from heterozygous (*t*/+) males [9,10]. Up to 99% of the offspring of *t*/+ males inherit the *t*-haplotype chromosome. A large body of evidence suggests that TRD is achieved by impairment of sperm motility by genetic variants expressed by the *t*-haplotype, termed distorters, in combination with *t*-sperm specific rescue of sperm motility by the responder, a dominant-negative protein kinase termed SMOK^TCR^ [11,12]. The effect of the distorters therefore provides access to understanding an important aspect of sperm motility control, successful competition by superior progressive movement. Four distorters have been identified so far, and all four represent genetic variants encoding regulators of Rho GTPases [13–15]. The most recently reported factor is the RAC-specific guanine nucleotide exchange factor TIAM2 [16]. The distorters thus suggest an important role of Rho signaling in the control of directed sperm movement.

Physiological analyses have provided strong evidence that motility control is impaired in sperm from mice carrying the *t*-haplotype. Homozygous *t*/*t* males produce sperm with almost no progressive motility [17] and abnormal flagellar curvature [18] resulting in male sterility [19]. Sperm from *t*/+ mice isolated either from the epididymis or from the female reproductive tract after mating, on the other hand, show decreased velocity, altered swimming trajectories and lower progressive motility compared to sperm from congenic +/+ mice [17,20,21]. It has been suggested that motile sperm in *t*/+ samples consists of two subpopulations, one more progressive and the other less progressive [18,22]. In combination with the high transmission ratio of the *t*-haplotype it was assumed that the advantage of *t*-sperm over +-sperm derived from *t*/+ males is based on superior motility. Consistent with this notion is the fact that the number of sperm reaching the oviducts of females mated with *t*/+ males was around half the number counted in females mated with +/+ males [23]. However, experimental evidence linking sperm motility properties with sperm genotype has been lacking.

Rho family proteins, namely RHOA, RAC1 and CDC42, are highly conserved small GTPases involved in signal transduction pathways controlling a variety of biological processes in eukaryotic cells, including actin polymerization and cell migration, in part *via* chemotaxis [24–28]. They act as molecular switches, by interconverting between active GTP-bound and inactive GDP-bound conformational states and are mainly regulated by GDP/GTP exchange factors (GEFs), GTPase-activating proteins (GAPs) and guanine nucleotide dissociation inhibitors (GDIs) [29]. The distorters involved in TRD we previously identified encode the GAP TAGAP, the GEF FGD2, the nucleoside diphosphate kinase NME3, and TIAM2, a GEF known to act specifically on RAC1 [13–15]. Therefore, we have proposed that RAC1 plays an important role in regulating sperm motility [16].

In this study we have investigated the role of RAC1 in mammalian sperm motility at the physiological level. Our data show that both enhanced and reduced RAC1 activity impairs sperm progressive movement and suggest that, within a sperm population, sperm with balanced RAC1 activity have an advantage in the race towards the egg cells. RAC1 thus plays a crucial role in controlling progressive motility and competitiveness of sperm.

## Results

### RAC1 inhibition affects sperm motility

**Fig 1:**
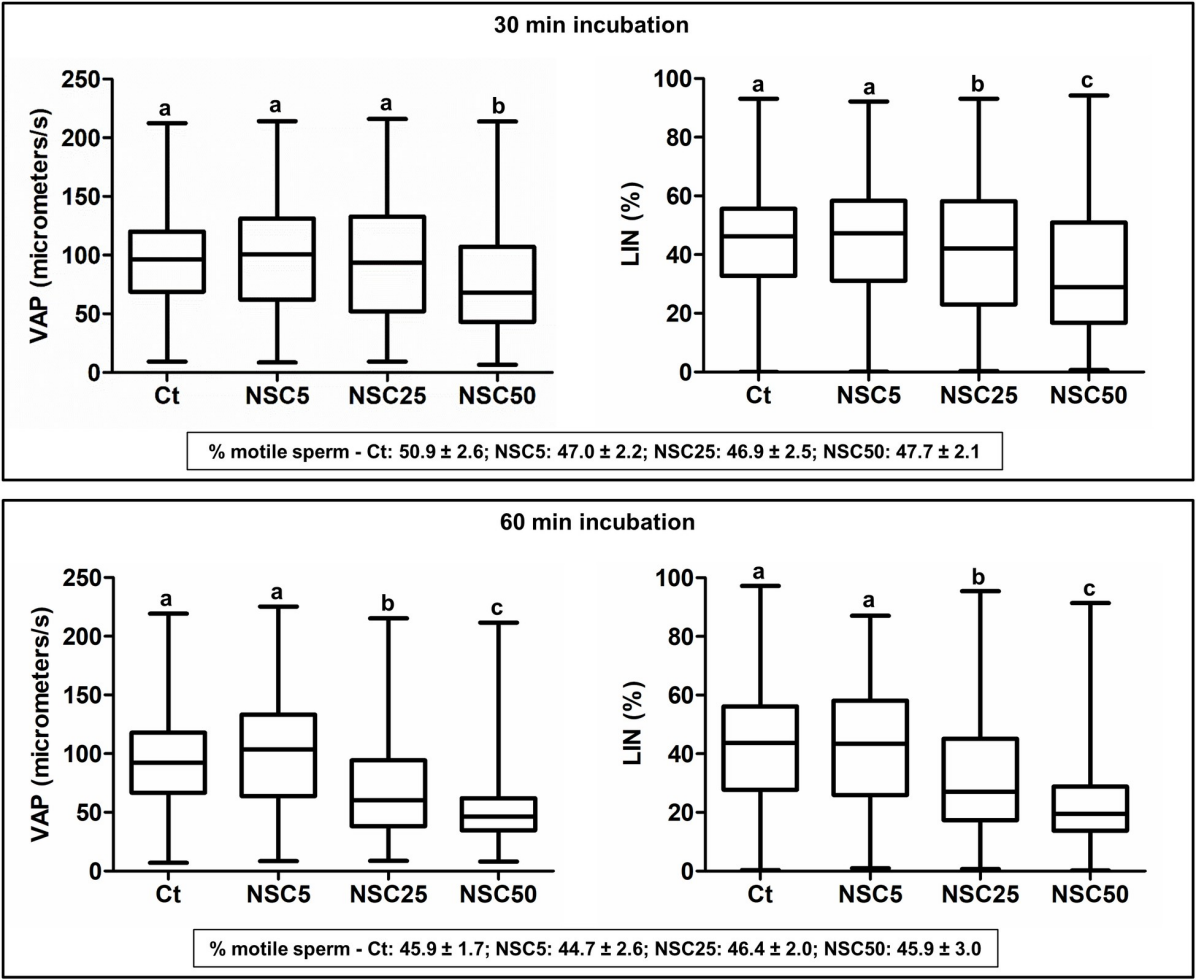
RAC1 inhibition affects motility parameters of wild type mouse sperm. Box plots (showing the median and first and third quartiles) with whiskers (from minimum to maximum) of average path velocity (VAP) and linearity (LIN) of sperm after incubation without (Ct: control) or with different concentrations of NSC23766 (NSC, μM) for 30 min (n = 2705, 2185, 2040 or 1581 sperm for Ct, NSC5, NSC25 or NSC50, respectively) and 60 min (n = 2178, 1597, 1431 or 1269 sperm for Ct, NSC5, NSC25 or NSC50, respectively). For each condition, the percentage of motile sperm (mean ± standard error, n = 11 samples) is also shown (bottom boxes). Statistically significant differences between treatments are indicated by distinct letters (*Ps* < 0.001).

A number of small molecule inhibitors targeting specific Rho-GTPases or their downstream effectors have been developed and extensively used to unravel the roles of Rho signaling in different somatic cell types [27]. To determine if Rac signaling is involved in sperm motility regulation, we have incubated wild-type sperm with the RAC1-specific inhibitor NSC23766 and checked if sperm motility parameters were affected, using an up-to-date computer assisted sperm motility analysis (CASA) system (S1 Fig). CASA outcomes clearly showed that RAC1 inhibition alters sperm motility and modifies the shape of sperm tracks, which tend to become circular, in a concentration- and time-dependent manner (S1–S4 Videos). RAC1 inhibition affected sperm velocity (resulting in lower values of average path velocity—VAP, straight-line velocity—VSL, and curvilinear velocity—VCL) and progressive motility (lower linearity—LIN, and straightness—STR), along with a decrease in the amplitude of the lateral head displacement (ALH) and an increase in beat cross frequency (BCF; Fig 1, S1 Table and S1 Dataset). RAC1 inhibition did not change the percentage of motile sperm in any of the experimental conditions tested (Fig 1, S1 Table and S2 Dataset). Similar results were obtained with the Rac inhibitor EHop-016, which also induced a decrease in sperm velocity and progressive motility (S1 Table and S3 Dataset), without having any effect on the percentage of motile sperm (S1 Table and S2 Dataset). Thus, motility parameters related to sperm velocity and progressive motility are controlled by Rac signaling.

### The *t*-haplotype also impairs sperm velocity and progressive motility

**Fig 2:**
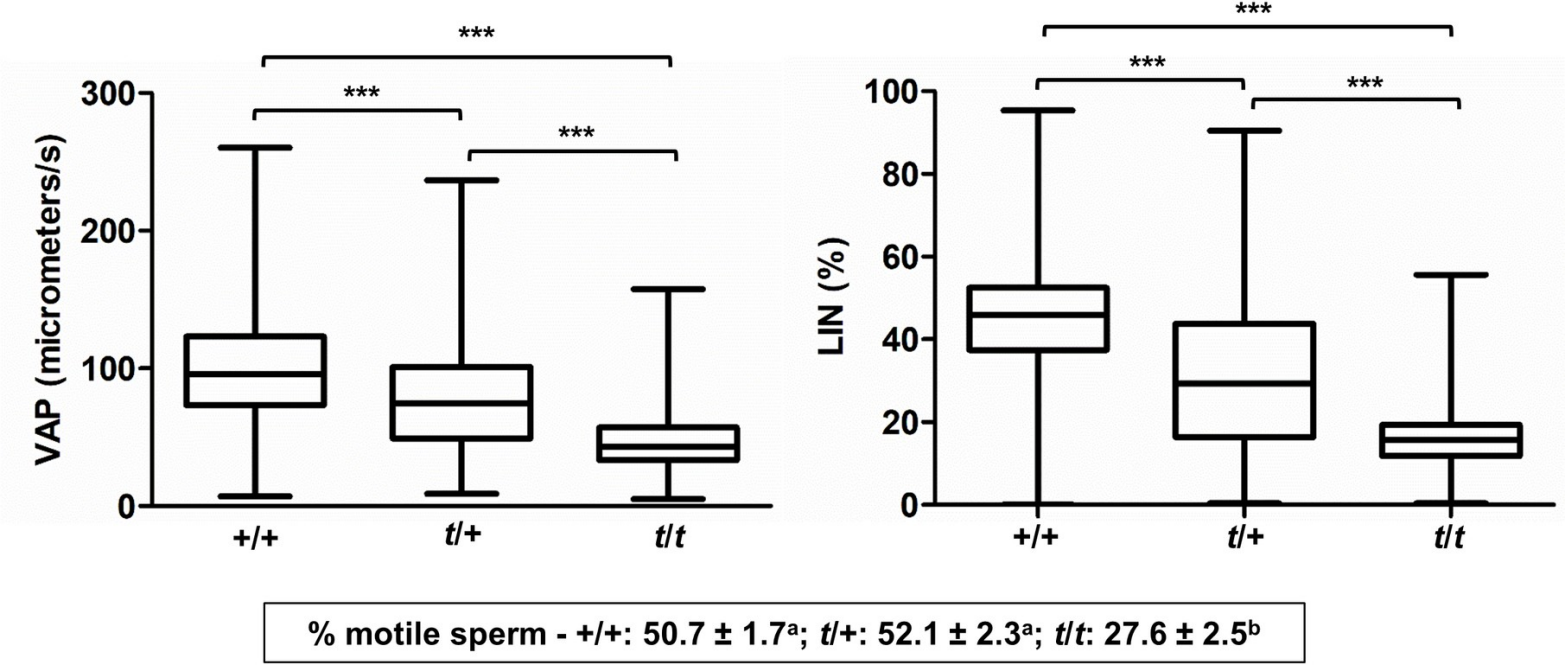
The *t-*haplotype impairs sperm progressive movement. Box plots (showing the median and first and third quartiles) with whiskers (from minimum to maximum) of average path velocity (VAP) and linearity (LIN) of sperm from wild type (+/+; n = 7151 sperm), heterozygous *t* (*t*/+; n = 8335 sperm) or homozygous *t* (*t*/*t*; n = 813 sperm) mice, immediately after sperm isolation. Asterisks indicate statistically significant differences between genotypes (***Ps < 0.001). The percentages of motile sperm (mean ± standard error; n = 15 +/+ samples, 15 *t*/+ samples and 15 *t*/*t* samples) are displayed in the bottom box (statistically significant differences between genotypes are indicated by distinct letters; *Ps* < 0.001).

Next, we asked if motility parameters affected by the *t*-haplotype resemble those affected by RAC1 inhibition in wild type sperm. We used CASA to compare sperm motility parameters in background-matched wild type (+/+), heterozygous *t* (*t*/+*)* and homozygous *t* (*t*^*w5*^*/t*^*w32*^*)* samples (S5–S7 Videos). Sperm from *t*/+ mice showed decreased velocity (lower VAP, VSL and VCL; *Ps* < 0.001) and progressive motility (lower LIN and STR; *Ps* < 0.001), compared to +/+ controls. The values were further reduced in sperm from *t*/*t* males (Figs 2 and S2 and S4 Dataset). Moreover, ALH was also decreased, while BCF was increased (*Ps* < 0.001). Therefore, the *t*-haplotype has a similar effect on sperm motility parameters as the RAC1 inhibitor NSC23766: both cause a decrease in VAP, VSL, VCL, ALH, STR and LIN, along with an increase in BCF.

### RAC1 activity is enhanced in *t*-sperm

**Fig 3:**
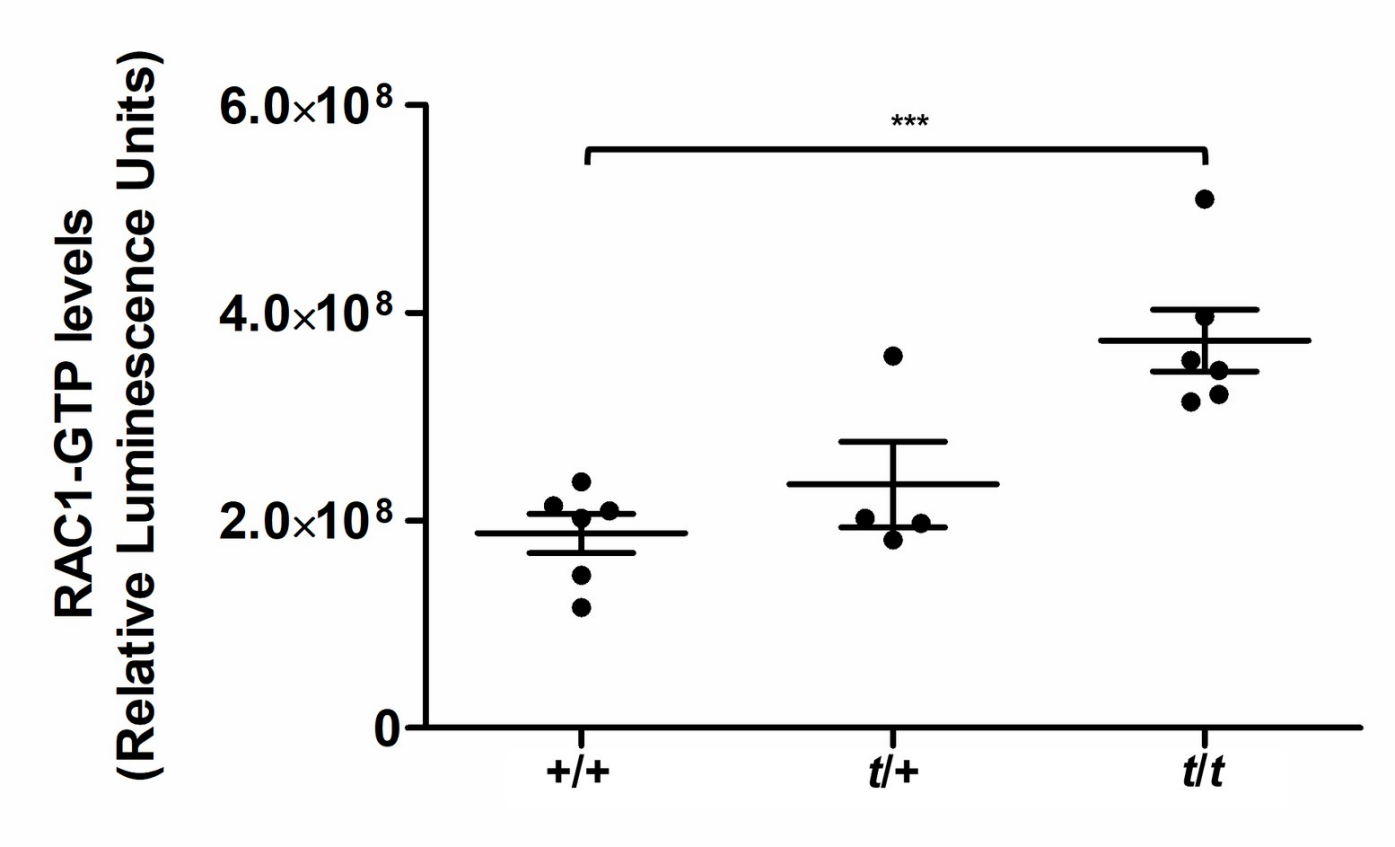
RAC1-GTP levels are strongly elevated in *t*-sperm. Scatter dot plots of RAC1-GTP levels in sperm from wild type (+/+; n = 6), heterozygous *t* (*t*/+; n = 4) or homozygous *t* (*t*/*t;* n = 6) mice (mean ± standard error is indicated). Each dot shows the mean of three technical replicates. Asterisks indicate a statistically significant difference between genotypes (***P < 0.001).

The above data suggested a possible link between aberrant RAC1 activity and impaired motility observed in sperm from males carrying the *t*-haplotype. In addition, genetic data previously have shown that expression of the RAC1 GEF TIAM2S from the *t*-haplotype is upregulated compared to wild-type, acts as distorter and enhances TRD in favor of the *t*-haplotype [16] suggesting that Rac1 signaling is increased in sperm derived from *t*/+ and *t*/*t* mice. To test if this is the case, we measured the levels of the active form of RAC1 (RAC1-GTP) in sperm samples. The data show that RAC1 activity indeed is strongly increased in *t*/*t* samples compared to +/+ (*P* < 0.001), while *t*/+ sperm on average show an intermediate level (Fig 3).

### *t*/+-derived sperm consist of two subpopulations with overlapping +*/*+ and *t*/*t* motility profiles

**Fig 4:**
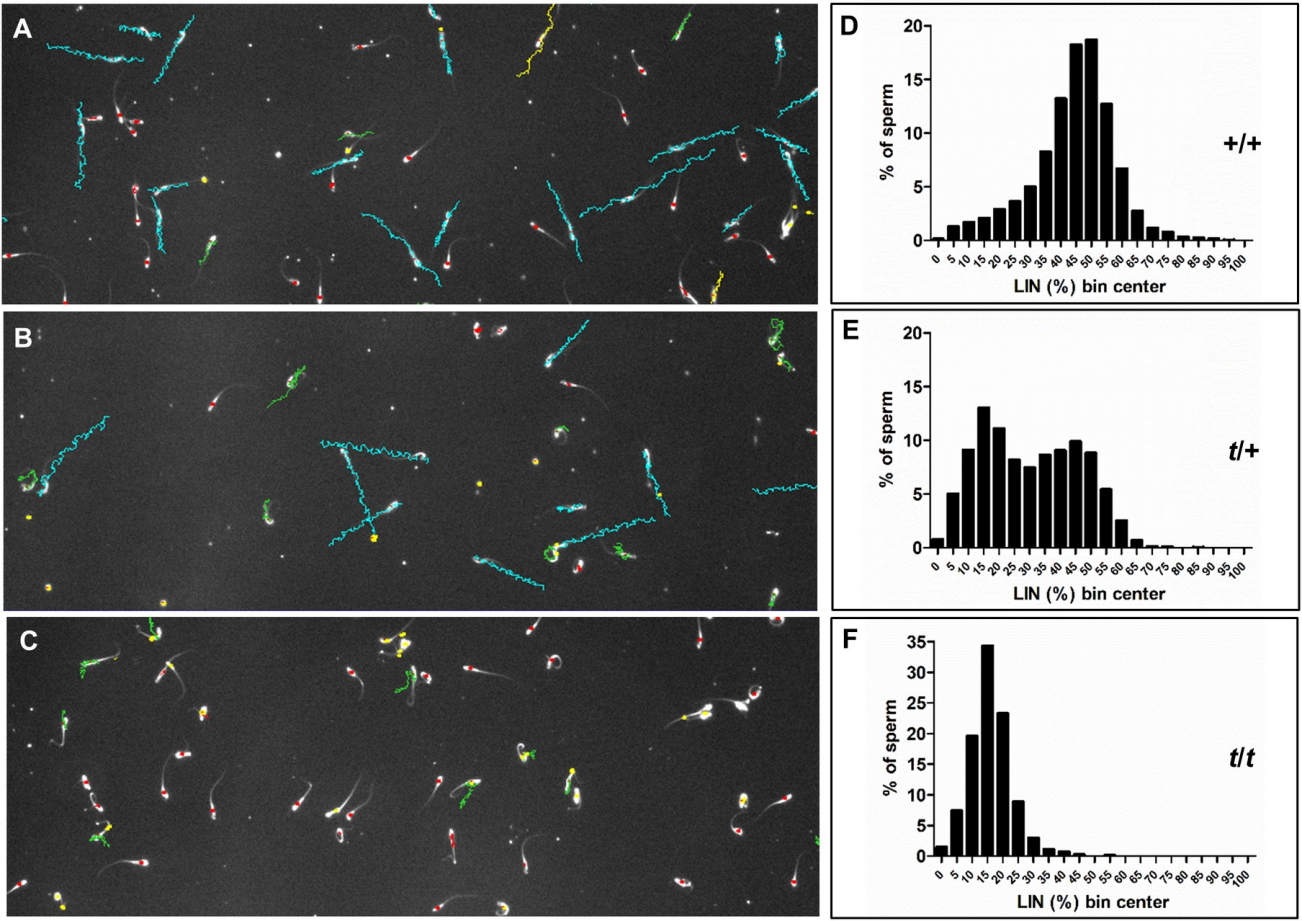
Motility tracks and linearity profiles of sperm from different genotypes. (A, B, C) Representative sperm tracks of +/+ (A), *t*/+ (B) and *t*/*t* (C) samples generated by CASA (computer assisted sperm motility analysis). Tracks color code: blue–progressive; green–less progressive; red–immotile (excluded from analysis); yellow–late tracks (recording started after the first 10 frames; excluded from analysis). (D, E, F). Histograms showing the frequency distribution of sperm over linearity (LIN; %) bins in +/+ (D; n = 7151 sperm), *t*/+ (E; n = 8335 sperm) and *t*/*t* (F; n = 813 sperm) samples (n = 15 samples each).

We observed sperm motility using CASA. The majority of motile sperm in +/+ samples displayed progressive movement (blue tracks; Fig 4A), while scarce motile sperm in *t*/*t* samples showed little progressive motility (green tracks; Fig 4C). Interestingly, in *t*/+ samples around half of the motile sperm showed progressive movement, while the other half moved less progressively (Fig 4B). These observations were corroborated by the frequency distributions of LIN, a measure of sperm progressive motility. As expected, in +/+ and *t/t* samples LIN follows normal “bell-shaped” distributions, with a strong offset of the histogram to the left in *t*/*t* sperm (Fig 4D and 4F and S2 Table). In contrast, the heterozygous (*t*/+) samples revealed a compound of overlapping “*t/t*-profile” and “+/+-profile” (Fig 4E), as reported previously [18]. Thus, the microscopic sperm tracks and the frequency distribution histograms show that *t*/+ derived sperm samples consist of two overlapping subpopulations of motile sperm with different progressive motility.

### Highly progressive sperm from *t*/+ males mostly carry the *t*-haplotype

**Fig 5:**
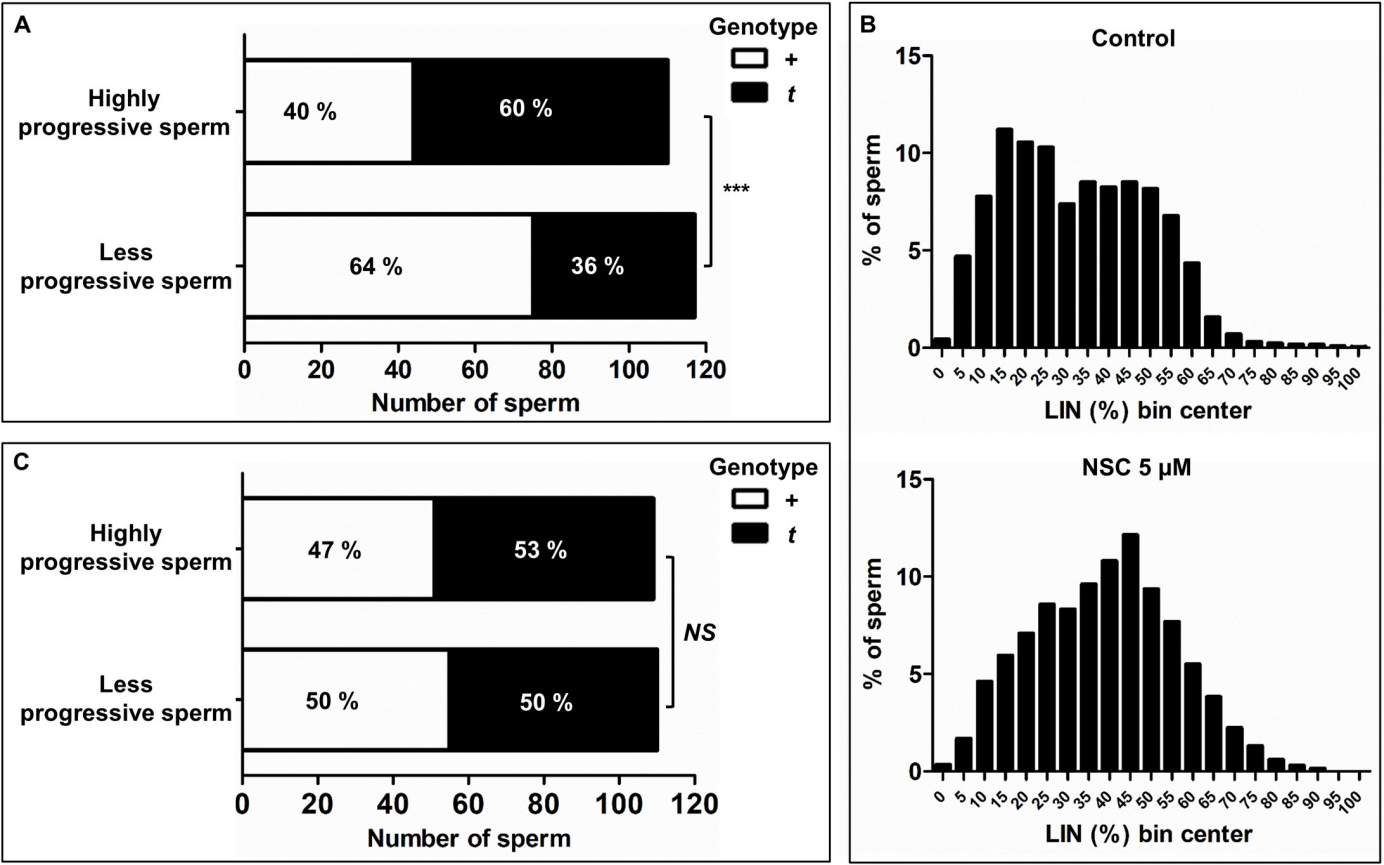
Effect of RAC1 inhibition on progressive motility of (+) sperm from heterozygous (*t*/+) mice. (A, C) Bar graphs showing the percentage of highly *vs*. less progressive sperm singly genotyped as *+* or *t* carriers. (A) Sperm isolated from *t/+* samples. (C) Sperm isolated from *t/+* samples incubated with 5 μM NSC23766 for 30 min. Asterisks indicate association (***P < 0.001) and *NS* (not significant) a random relation between progressiveness and *t* genotype of sperm. (B) Histograms showing the frequency distribution of sperm over linearity (LIN; %) bins in *t*/+ samples (n = 7) incubated without (controls; upper graph; n = 2019 sperm) or with 5 μM NSC23766 (lower graph; n = 2306 sperm) for 30 min.

If high RAC1-GTP causes low progressiveness one would expect that among *t*/+ derived sperm the *t*-haplotype is more frequently found in less progressive sperm. However, the *t*-haplotype promotes its own transmission to the next generation suggesting that *t*-sperm should move more progressively than +-sperm. To determine which assumption holds true we used micro-pipetting under a stereo microscope to isolate highly or less progressive single sperm cells (for details see Materials and Methods). Genotyping of more than 100 single sperm cells of each phenotype by PCR (S3 Fig) revealed that highly progressive sperm more frequently contained the *t*-haplotype, while less progressive sperm are enriched for the wild type chromosome (Fig 5A; *P* = 0.0005). Thus, our data, for the first time, demonstrate that with respect to progressive movement, among sperm derived from *t*/+ males *t*-sperm are indeed superior to +-sperm.

### RAC1 inhibition rescues progressive motility in +-sperm derived from *t*/+ males

The above data suggest that the *t*-haplotype is able to trigger high RAC1-GTP levels in +-sperm derived from *t*/+ males, and thereby impair progressive motility in +-sperm, while *t*-sperm are protected and move more progressively. If that were the case, RAC1 inhibition should rescue progressive motility in +-sperm among *t*/+ sperm samples. Indeed, after a short (i.e., 30 min) incubation with low concentrations of NSC23766 (5 μM), the sperm motility trajectories of *t*/+ samples resembled those of +/+ samples (S8 and S9 Videos). This microscopic observation was confirmed by frequency distribution histograms of sperm linearity (Fig 5B), that change from a bimodal distribution (typical for *t*/+ samples) in controls to an almost normal distribution typical for +/+ samples (compare with Fig 4D) in the presence of NSC23766 (S2 Table). These data suggested that RAC1 inhibition at low NSC concentrations at least partially rescues the motility of the low-LIN subpopulation of sperm derived from *t*/+ males (Fig 5B). Progressive motility (LIN and STR) and, to a slight extent, velocity (VAP and VSL) were improved (S1 Table, S4 Fig and S5 Dataset). Improved progressive motility of *t*/+ samples was also achieved by the incubation with low concentrations of EHop-016 (S1 Table, S5 Fig and S6 Dataset). However, at high NSC23766 concentrations this rescue effect was reversed, and progressive motility was again impaired as observed in +/+ samples (S1 Table, S4 Fig and S5 Dataset).

To obtain further evidence that low RAC1 inhibition indeed rescued the +-sperm subpopulation among *t*/+ samples we isolated highly or less progressive single sperm cells from NSC-treated *t*/+ samples by micro-pipetting (Fig 5C). Genotyping of over 100 sperm of each phenotype showed that sperm genotype and progressive motility were unrelated. Highly progressive sperm contained the *t-*haplotype only slightly, but insignificantly more often. Less progressive sperm showed equal distribution of *t*- and +-chromosomes. Thus, RAC1 inhibition by NSC indeed rescued progressive motility in +-sperm among *t*/+ samples.

We then asked if a reduction of RAC1 activity by NSC23766 might also have an effect on the progressive movement of sperm from *t*/*t* males. Our data show that treatment with NSC23766 for 30 min slightly improved sperm linearity and increased the percentage of motile sperm at higher NSC23766 concentrations (25–50 μM) (S3 Table and S7 Dataset).

The combined data suggest that the progressive motility of sperm requires an optimal level of RAC1-GTP. High RAC1-GTP levels, as observed in *t*/*t* sperm samples, strongly impair sperm progressive movement, and low RAC1-GTP, as achieved by high NSC23766 concentrations, also impairs velocity and progressive motility of +/+-derived sperm. In *t*/+ sperm, the *t*-haplotype is able to trigger elevated RAC1-GTP levels, which impair progressive motility in +-sperm, while sperm progressive motility in *t*-sperm is not affected due to rescue by the dominant-negative kinase SMOK^TCR^ [10,11]. Taken together, our data provide strong evidence that the Rac1 signaling pathway acts as regulator of progressive movement in mouse sperm.

### RAC1 inhibition also modulates the motility of bull sperm

**Fig 6:**
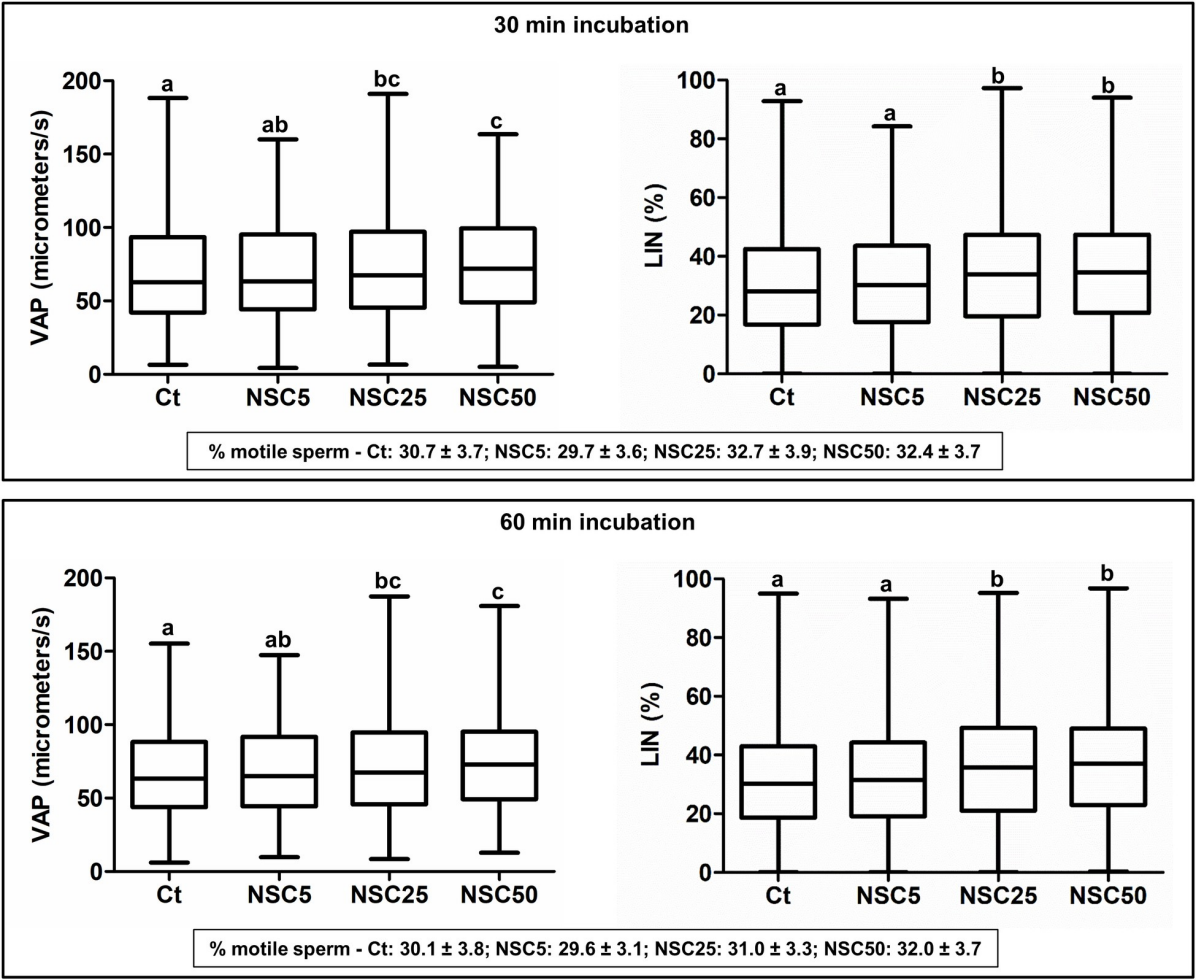
Effects of RAC1 inhibition on motility parameters of bull sperm. Box plots (showing the median and first and third quartiles) with whiskers (from minimum to maximum) of average path velocity (VAP) and linearity (LIN) of sperm incubated without (Ct: control) or with different concentrations of NSC23766 (NSC, μM) for 30 min (n = 2186, 2224, 2116 or 2493 sperm for Ct, NSC5, NSC25 or NSC50, respectively) and 60 min (n = 2128, 2092, 2156 or 2572 sperm for Ct, NSC5, NSC25 or NSC50, respectively). For each condition, the percentage of motile sperm (mean ± standard error, n = 7 samples) is also shown (bottom boxes). Statistically significant differences between treatments are indicated by distinct letters (*Ps* < 0.001).

Finally, we asked whether Rac1 signaling is also involved in sperm motility regulation in another mammalian species. We analyzed the effects of NSC23766 treatment on bull sperm, and show that RAC1 inhibition also affects bull sperm motility parameters (Fig 6, S4 Table and S8 Dataset). However, in contrast to wild type mouse sperm, NSC23766 induces statistically significant increases in sperm velocity (higher values of VAP, VSL and VCL) and progressive motility (increased LIN and STR), along with a decrease in beat frequency (lower BCF values), in a concentration-dependent manner. Similar results were obtained with the RAC inhibitor EHop-016 (S4 Table and S9 Dataset). Thus, RAC1 inhibition improved the motility of ejaculated bull sperm. The data suggest that RAC1 might be a common regulator of sperm motility in mammals.

## Discussion

Here we present strong evidence that RAC1 plays an important role in controlling progressive movement in mouse sperm. In addition, our data reveals that progressive motility requires fine tuning of RAC1 activity. In conjunction with TRD triggered by the *t*-haplotype, our data suggest that unsuitable levels of RAC1 activity can impair directed movement in individual sperm and thus their ability to compete successively in the race for egg cells prior to fertilization. Moreover, excessive RAC1 activity is involved in rendering sperm immotile causing male sterility.

Although it has long been assumed that *t*-haplotype TRD is triggered by differences in the motility of *t*- and +-sperm derived from *t*/+ heterozygous males, this has never been proven experimentally. Here, by genotyping single sperm from *t*/+ mice isolated according to motility patterns (highly progressive *versus* less progressive), we have validated the hypothesis that *t*-sperm overall move better forward than +-sperm. This differential ability explains the advantage of *t*-sperm over +-sperm in reaching and fertilizing egg cells, thereby promoting the transmission of the *t*-haplotype to the next generation. Interestingly, though sperm from *t*/*t* males become immotile very quickly and *t*/*t* males are consequently sterile, this deficiency is turned into an advantage for *t*-sperm among sperm from *t*/+ males. This truly amazing trick of the *t*-haplotype is based on the rescuing effect of the responder, the dominant-negative kinase SMOK^TCR^ [11]. The latter has been proposed to counterbalance the “poisoning” effect of the distorters encoding regulators of Rho GTPases such as the RAC1-specific GEF TIAM2 [16]. We have previously shown that overexpression of TIAM2S from a transgene construct promotes the transmission of the *t*-haplotype from *t*/+ males. Our current data therefore support our hypothesis that TIAM2S, by increasing the activity of RAC1, contributes to the impairment of sperm motility. The latter occurs in all sperm from *t*/+ males, while the rescuing activity, SMOK^TCR^, is restricted to *t*-sperm [30], and this is the reason why specifically *t*-sperm can compensate for the deregulated Rho signaling induced by distorters and regain proper forward movement. The fact that lowering RAC1 activity in sperm from *t*/+ males by the inhibitor NSC23766 equalizes the motility behavior of (+)-sperm and *t*-sperm confirms the conclusion that indeed +-sperm cells from *t*/+ males are compromised and further emphasizes the important role of RAC1 in progressive sperm movement and TRD (S6 Fig). *Rac1* also is the first gene located outside the *t*-haplotype shown to be involved in TRD.

How are Rho GTPase signaling and the sperm motility kinase SMOK, which are crucial for TRD, linked to the axoneme, which generates motion? The dominant-negative nature of the responder, SMOK^TCR^, previously led us to propose that the distorters control SMOK through an activating and an inhibitory pathway, and that SMOK is a central regulator of flagellar movement [11,14–16]. Our data confirm that RAC1, and thus Rho signaling is crucial for progressive forward movement of sperm, and link the high success rate of *t*-sperm from *t*/+ males in fertilization resulting in TRD, with the control of sperm progressive movement by RAC1. Previous reports have shown that sperm can be guided *via* chemotaxis to the egg cells and that follicle cells may provide the chemotactic signal [31]. There is also a large body of evidence demonstrating an important role of RAC1 in cell migration as well as in chemotaxis of somatic cells [24,25,28,32]. Therefore, it is quite suggestive that RAC1 might be involved in directing sperm along the gradient of a chemoattractant towards the egg cells *via* chemotaxis. This would provide a plausible link between the central role of RAC1 in the control of sperm progressive movement, the (SMOK activating) distorter pathway, and the high fertilization success of *t*-sperm from *t/+* males. It would also imply that Rho GTPase signaling acts between an unknown chemotactic sensor (receptor) in the plasma membrane, and SMOK. It is known that dynein arm phosphorylation by kinases activates dynein ATPases, and the hydrolysis of ATP is converted into force effecting microtubular sliding in the axoneme; and SMOK might be one of the kinases involved in dynein control. Dephosphorylation reverses this process. Both the cyclic AMP/protein kinase A (cAMP/PKA) pathway and calcium signaling are at the hub of motility control, but many more interconnected regulatory pathways are involved (for reviews see [1,2,4,33,34]). It is quite likely that these pathways are mainly implicated in generating motion, while according to our data Rho signaling and SMOK are involved in directing (“steering”) sperm cells.

Enhanced activation (+-sperm from *t/+* mice) as well as inhibition (NSC23766 or EHop-016 in +/+ mice) of RAC1 result in reduced sperm progressive motility, emphasizing the requirement of Rac fine-tuning for proper forward movement of mouse sperm. Similarly, it has been shown that a small change in Rac activity alters the directionality of cell migration in human fibroblasts and epithelial cells, among other somatic cells, and that either too high or too low Rac activities inhibit cell migration [35]. Moreover, RAC1 was suggested to have a dual role in regulating mesenchymal cell migration and invasion, by driving two signaling cascades, one promoting and one inhibiting cellular movement [36].

Our data show that the velocity and progressive motility of bull sperm were improved by treatment with the RAC1 inhibitors NSC23766 and EHop-016, suggesting that RAC1 is slightly overactive in ejaculated bull sperm, which might have been induced by the cryopreservation process [37,38]. However, since the semen donors were individual males of a particular breed, natural variation giving rise to slightly non-optimal Rac activity cannot be excluded. The results suggest that RAC1 activity modulates sperm swimming behavior not only in mice, but also in other mammals.

It is well established that Rac1, RhoA and Cdc42 are expressed in mammalian sperm head and tail [39–41]. Their expression in the head has mainly been studied in the context of actin polymerization during capacitation and acrosome reaction control [42–47]. But there are also studies pointing to a role of Rho GTPases in the sperm tail, related to motility control. For instance, long-time incubation of bovine sperm with a Rho inhibitor resulted in motility decline [48]. Moreover, rhophilin, a Rho small GTPase-binding protein, and its binding partner ropporin, are expressed in the sperm tail principal piece and were suggested to be motility modulators [49,50]. Interestingly, mice lacking either ropporin 1 or ropporin 1-like protein showed altered sperm motility parameters, with lower velocities, lower LIN and STR and higher BCF [51], alterations that resemble the ones we observed in mice expressing the *t*-haplotype. The involvement of Rho signaling in human sperm motility regulation was also suggested by results of proteomic comparisons between normozoospermic and asthenozoospermic (i.e., presenting with low sperm motility) cohorts [52,53]. It will be interesting to see if Rac also controls human sperm, and if any forms of male infertility caused by impaired sperm motility in man may be related to improper Rac activity.

Although cell motility control by Rho GTPases is usually associated with actin cytoskeleton regulation, Rho GTPases are also known to influence microtubule organization and dynamics in somatic cells [54–57]. The (concomitant) involvement of actin regulation may also play a role in the molecular organization of the flagellar structures implicated in sperm motility [58]. And in fact, actin polymerization modulators were shown to induce sperm motility defects in different mammalian species, including guinea pig [59], human [60,61] and macaque where a RhoA signaling mechanism seems to be involved [62]. Moreover, actin reorganization may also participate in the acquisition of sperm motility during epididymal maturation, through pathways involving both Rac1 and Cdc42 [63].

## Materials and methods

### Ethics statement

Animal usage for organ dissection was approved by the Animal Welfare Committee of the Max Planck Institute for Molecular Genetics (under license number T0279/16 of the LaGeSo Berlin).

### Reagents and medium

All reagents were purchased from Sigma-Aldrich (St. Louis, MO, USA) unless otherwise stated.

Sperm isolation, processing and motility analyses were done using fresh and filtered supplemented Krebs-Ringer (sKR) medium [93.7 mM NaCl, 4.7 mM KCl, 1.2 mM KH_2_PO_4_, 1.2 mM MgSO_4_, 1.7 mM CaCl_2_, 5.6 M glucose, 0.33 mM sodium pyruvate, 21 mM sodium lactate, 25 mM NaHCO_3_, 10 mM HEPES, 1% (w/v) bovine serum albumin, 100 units/ml potassium penicillin and 0.1 mg/ml streptomycin sulfate, pH 7.4], pre-equilibrated at 37°C and 5% CO_2_. This medium mimics the physiological conditions of the mammalian female reproductive tract and induces sperm capacitation.

#### Mice

Sexually mature male congenic mice (i.e., at least 8 weeks old) with different *t* genotypes were used in this study: a) wild-type (+/+; C57BL/6J), b) heterozygous *t* (*t*/+; mice carrying one of the naturally occurring complete *t*-haplotypes—*t*^*w5*^ or *t*^*w32*^- maintained for > 100 generations on the C57BL/6J genetic background—in heterozygosity), c) homozygous *t* (*t*/*t*; mice carrying *t*^*w5*^ and *t*^*w32*^ in compound heterozygosity). All comparisons between genotypes were done using age-matched animals.

#### Bull semen

Cryopreserved semen samples from 7 Holstein bulls were provided by Rinderbesamungs Genossenschaft Memmingen e.G. (Memmingen, Germany).

### Mouse epididymal sperm isolation and preparation

Mice were euthanized by cervical dislocation and epididymides were isolated, cleaned from adipose and connective tissues, and rinsed in sKR medium. Cauda epididymides free of large blood vessels (carefully extracted with tweezers) were transferred to 1 ml sKR medium and nicks were made with a needle, while applying small pressure with tweezers, to induce sperm release. This was followed by 10 min incubation at 37°C, 5% CO_2_, to allow sperm to swim-out, after which the tissue was removed. To discard any remaining tissue pieces, suspensions were centrifuged for 1 min, at 100 x *g* and clean suspensions were gently mixed by swirling. Wide bore pipette tips were used for all sperm pipetting.

### Bull sperm preparation

Cryopreserved semen samples were thawed in a water bath at 37°C and centrifuged for 5 min at 800 x *g*. Sperm pellets were washed and resuspended in sKR medium.

### RAC1 inhibition

In order to study the putative effects of RAC1 inhibition in sperm motility, we took advantage of available pharmacological tools. Two well-characterized RAC1 inhibitors were selected (NSC23766 and EHop-016; Tocris Bioscience, Bristol, UK) and tested in the ranges of concentrations used by others in different mammalian cells. NSC23766 and EHop-016 are cell-permeable small molecule inhibitors that prevent RAC1 activation by RAC1-specific GEFs, with an IC_50_ of 50 μM and 1 μM, respectively [64–66]. NSC23766 inhibits RAC1 specifically, while EHop-016 inhibits both RAC1 and RAC3 (as well as CDC42 at concentrations > 5 μM). Worth mentioning, and as observed by others for NSC [45], the inhibitors do not affect mouse sperm viability in the concentrations and incubation times used.

Small Petri dishes containing sKR medium were prepared in the absence (control) or presence of increasing concentrations of NSC23766 (5 μM, 25 μM, 50 μM) or EHop-016 (1 μM, 2.5 μM, 5 μM). Stock solutions with distinct concentrations of the drugs were prepared, so that equal volumes of drugs were added in the different conditions. The same volume of water (NSC) or DMSO (EHop) was added to controls. Sperm samples were added to each Petri dish (final concentration of around 2 x 10^5^ cells/ml) at successive time points and were incubated at 37°C, 5% CO_2_. Motility analysis was performed by CASA as described next, after 30 min and 60 min incubation. Data from different samples were combined (n = 4 to 11, depending on the experiment), so that around 1000–2000 sperm tracks were analyzed for each experimental condition. For *t*/*t* mouse sperm the number of tracks analyzed ranged from 339 to 495.

### Computer assisted sperm motility analysis (CASA)

CASA uses serially captured digital images of motile sperm heads to reconstruct individual trajectories (sperm tracks) and compute parameters defining sperm kinematics [67,68], namely describing sperm speed, progression and trajectory shape (S1 Fig). Three sperm trajectories are computed for each sperm: the actual trajectory (called curvilinear path), the average trajectory (average path) and a straight-line path from the first to the last detected sperm head position (straight-line path). Based on these paths, three velocities (μm/s) are defined: curvilinear velocity (VCL), average path velocity (VAP) and straight-line velocity (VSL). The deviation of a sperm head from its average path is called amplitude of lateral head displacement (ALH; μm). And the average rate at which the curvilinear path crosses the average path is termed beat cross frequency (BCF; number per second; a derivation of the frequencies of rotation of the head and of flagellar beat). The linearity of the curvilinear path and of the average path are given by LIN (linearity = VSL/VCL; %) and STR (straightness = VSL/VAP; %), respectively.

Samples were diluted to a concentration of around 2 x 10^5^ sperm/ml using sKR medium and incubated at 37°C, 5% CO_2_ for 5 min. Sperm motility was analyzed by the CASA system CEROS II Animal Motility (software version 1.7.1; Hamilton Thorne, Beverly, MA, USA), using a BA310 microscope (Motic, Beijing, China), equipped with a MiniTherm stage warmer and a JAI CM-030-GE CCD camera. Sperm tracks (1.5 s) were captured at 37°C with a 4x dark-field objective, by recording 90 frames at a 60 Hz frame capture speed. The camera was set to 14 ms exposure, with 600 gain. Kinematics settings were: cells counted as progressive if STR > 50% and VAP > 50 μm/s; slow cells VAP and VSL cut-offs 10 μm/s and 0 μm/s, respectively; static VSL and VAP cut-offs of 0 μm/s. Pre-warmed Leja slides (100 μm depth; Leja Products B.V., Nieuw Vennep, The Neatherlands) were used and the capillary correction was set to 1.3. Slides were cleaned with lens-cleaning paper and each chamber was filled with 25 μL sperm suspensions, by capillary action. For each sample analyzed, tracks and kinematic parameters of sperm in several non-overlapping fields were recorded, covering the whole viewable area of the two chambers within one slide. Tracks from immotile sperm or from motile sperm with less than 45 points (half the number of total frames) were not included in the analyses.

#### Mouse sperm

For illumination, maximum and minimum photometer values were set to 42 and 37, respectively. For cell detection, the following settings were used: 90% maximum elongation, 1% minimum elongation, 135 minimum head brightness, 200 μm^2^ maximum size head, 20 μm^2^ minimum size head, 52 minimum tail brightness, 10 minimum tail brightness auto offset, using minimum tail brightness manual mode.

For the comparison of sperm motility parameters between genotypes, 15 +/+, 15 *t*/+ and 15 *t*/*t* mice were used and data from the different animals were combined for each genotype (total number of sperm analyzed: 7151 +/+; 8335 *t*/+; 813 *t/t*).

#### Bull sperm

The CASA setup used for bull sperm analysis was similar to the one used for mouse sperm, except for the following settings: maximum and minimum photometer values were set to 48 and 43; 98 minimum head brightness, 47 minimum tail brightness and 10 μm^2^ minimum size head were used for cell detection.

#### Total sperm motility

The percentage of motile sperm (i.e., showing any kind of motility) was determined by scanning the recorded videos. At least 200 sperm, from at least four different videos (i.e., four non-overlapping microscopic chamber fields) were counted per sample.

### RAC1-GTP levels

Sperm RAC1-GTP levels (a proxy for RAC1 activity) were compared between +/+ (n = 6), *t*/+ (n = 4) and *t*/*t* (n = 6) mice by using the luminescence-based G-LISA Rac1 activation assay biochem kit (Cytoskeleton, Inc., Denver, CO, USA), according to the manufacturer´s guidelines. Samples were washed in Dulbecco’s Phosphate Buffered Saline (DPBS; Lonza, Basel, Switzerland) and sperm pellets were used to prepare lysates. A preliminary experiment was performed in order to check that the assay was in the linear range. Extracts were equalized to 0.4 mg/ml total protein. Samples were run in triplicates and readings were done using a GloMax multi-detection system (Promega, Madison, WI, USA). Only triplicate values with low variance were accepted.

### Isolation and genotyping of single mouse sperm with different progressiveness

Two sets of experiments were performed using heterozygous *t* (*t*/+) samples: 1) immediately after sperm isolation; 2) after 30 min incubation with 5 μM NSC. Ten μl sperm suspensions (diluted 1500-3000X in sKR medium) were dropped in small Petri dishes and covered with mineral oil. Sperm were observed with a Leica M205 C stereo microscope using Rottermann Contrast (RC; Nußloch, Germany) and the swimming pattern of individual sperm were carefully examined. Motile sperm a) highly progressive and b) less progressive were individually isolated using pulled glass capillary tubes with metal holder and filtered tubing controlled with a mouthpiece. To distinguish highly progressive and less progressive sperm we used several videos of heterozygous *t* (*t*/+) samples recorded with the CASA system from which the marked sperm tracks were omitted (S10 Video) as training set. By viewing the same sperm tracks with color code (i.e., blue for highly progressive and green for less progressive sperm), we could compare our classifications with those set by CASA. We then practiced observing sperm moving in drops in small Petri dishes for longer time periods (the videos recorded by CASA are short) using a stereo microscope. At last, only sperm that were clearly distinct in motility, either evidently highly progressive (straight trajectory) or less progressive (sinuous trajectory) were isolated and subjected to PCR analysis. For each sample (n = 10 for experiment 1; n = 11 for experiment 2), around 20 highly progressive sperm, 20 less progressive sperm and 20 negative controls (i.e., culture medium without any sperm—to exclude false positive results caused by amplicon carryover contamination, a common issue in single cells PCR) were isolated. Single sperm DNA extraction and amplification were done using the REDExtract-N-Amp Tissue PCR Kit, according to the manufacturer´s indications, with small modifications. Single sperm/negative controls were incubated in 4 μl extraction buffer supplemented with 40 mM DTT for at least 1h at room temperature. After 3 min incubation at 95°C, 4 μl neutralization solution was added and samples were stored at 4°C. For positive controls, sperm samples from +/+, *t*/+ and *t*/*t* mice were used. Single sperm were genotyped by 40 μl PCR using primers Vil2-L and Vil2-R (TCATGGACCAACACAAGCTC and CACAAAACTGAAATCTCCCTCTC; MGI accession ID: 3033374) with the following cycling conditions: initial denaturation at 94°C for 3 min, 40 cycles with denaturation at 94°C for 30 s, annealing at 57°C for 35 s and extension at 72°C for 30 s, and final extension at 72°C for 2 min. The resulting PCR-amplified fragment displays a length polymorphism distinguishing the wild-type (228 bp fragment) and *t*-haplotype (195 bp fragment) proximal region of chromosome 17. PCR products were resolved on 4% agarose gels, and product size confirmed against a 100 bp plus DNA ladder (Thermo Fisher Scientific, Waltham, MA, USA).

The results of the different samples were pooled, reaching a total of 226 (experiment 1; 109 highly progressive sperm and 117 less progressive sperm) and 219 (experiment 2; 109 highly progressive sperm and 110 less progressive sperm) unequivocally genotyped sperm.

### Statistical analyses

Statistical analyses were done using GraphPad Prism version 5.03 for Windows (GraphPad Software, San Diego, CA, USA). The normality of numerical data distributions was tested using D’Agostino and Pearson omnibus normality test and the subsequent tests were chosen accordingly. Sperm kinematic parameters were compared (between *t* genotypes, and between drug treatments and controls) using Kruskal-Wallis test and Dunn´s multiple comparison test. The percentages of motile sperm in mouse samples of different *t* genotypes were compared using one-way analysis of variance (ANOVA) and Tukey´s multiple comparison test. Repeated measures ANOVA and Tukey´s multiple comparison test was used to compare the percentages of mouse or bull motile sperm in drugs experiments (pairing was significantly effective in all cases). Individual values for RAC1 activity were obtained by calculating the mean of the triplicates and samples were compared by one-way ANOVA and Tukey´s multiple comparison test. Categorical data (sperm progressiveness over genotype contingency tables) were analyzed by Fisher´s exact test. *P* values < 0.001 were considered significant.

## Supporting information

S1 Table Sperm motility and kinematic parameters in wild type (+/+) and heterozygous *t* (*t*/+) mouse samples incubated in the absence (controls) or presence of the RAC1 inhibitors NSC23766 or EHop-016 for 30- and 60-min. The percentage of motile sperm is given as the mean ± standard error. Kinematic parameters are expressed as median (with 25^th^-75^th^ percentiles). For each parameter, within each genotype, statistically significant differences between treatments are indicated by different letters (*Ps* < 0.001).
(TIF)

S2 Table Frequency distribution over sperm linearity bins. Percentages of sperm in each linearity (LIN) BIN and cumulative percentages of more progressive sperm (sum indicated in red; dashed line indicates the transition between the +*/*+ and *t/t* profile) are shown. (Left side) Comparison between sperm from wild type (+/+; n = 7151), heterozygous *t* (*t*/+; n = 8335) and homozygous *t* (*t*/*t*; n = 813) mice. (Right side) Comparison between sperm from *t/+* samples incubated without (control–Ct; n = 2306 sperm) or with 5 μM NSC23766 (NSC5; n = 2019 sperm) for 30 min.
(TIF)

S3 Table Sperm motility and kinematic parameters in homozygous *t* (*t*/*t*) mouse samples incubated in the absence (controls) or presence of the RAC1 inhibitor NSC23766 for 30 minutes. The percentage of motile sperm is given as the mean ± standard error. Kinematic parameters are expressed as median (with 25^th^-75^th^ percentiles). Statistically significant differences between treatments are indicated by different letters (*Ps* < 0.01).
(TIF)

S4 Table Sperm motility and kinematic parameters of bull samples incubated in the presence or absence (controls) of the RAC1 inhibitors NSC23766 or EHop-016 for 30- and 60-min. The percentage of motile sperm is given as the mean ± standard error. Kinematic parameters are expressed as median (with 25^th^-75^th^ percentiles). Statistically significant differences between treatments are indicated by distinct letters (*Ps* < 0.001).
(TIF)

S1 Fig Computer Assisted Sperm Motility Analysis (CASA) parameters describe sperm speed, progression and trajectory shape. Schematic drawing explaining the kinematic parameters computed by CASA. Three trajectories are established: curvilinear path (real trajectory), average path, and linear path (straight line trajectory). Three velocities (μm/s) are calculated based on these paths: curvilinear velocity (VCL), average path velocity (VAP) and straight-line velocity (VSL). The amplitude of the lateral head displacement (ALH; μm) is the deviation of a sperm head from its average path. Beat cross frequency (BCF; number per second) is the average rate at which the curvilinear path crosses the average path (a derivation of the frequencies of rotation of the head and of flagellar beat). The linearity of the average path is given by straightness (STR = VSL/VAP; %) and the linearity of the curvilinear path is called linearity (LIN = VSL/VCL; %).
(TIF)

S2 Fig Additional motility parameters of sperm from mice with different *t* genotypes. Box plots (showing the median and first and third quartiles) with whiskers (from minimum to maximum) of straight-line velocity (VSL; A), curvilinear velocity (VCL; B), amplitude of the lateral head displacement (ALH; C), beat cross frequency (BCF; D) and straightness (STR; E) of sperm from wild type (+/+; n = 7151 sperm), heterozygous *t* (*t*/+; n = 8835 sperm) and homozygous *t* (*t*/*t*; n = 813 sperm) mice (n = 15 mice for each genotype), immediately after sperm isolation. Asterisks indicate statistically significant differences between genotypes (***Ps < 0.001).
(TIF)

S3 Fig Genotyping of single sperm by PCR. Gel image of DNA fragments amplified by PCR of *t/+*, single *+*-sperm or *t*-sperm (isolated by micro-pipetting of highly or less progressive sperm), and separated by agarose gel electrophoresis (+ band: 228 bp; *t* band: 195 bp). *t*/+ sperm and cell-free culture medium served as positive and negative controls, respectively.
(TIF)

S4 Fig Effects of RAC1 inhibition on motility parameters of sperm from heterozygous *t* (*t*/+) mice. Box plots (showing the median and first and third quartiles) with whiskers (from minimum to maximum) of average path velocity (VAP) and linearity (LIN) of sperm incubated without (controls–Ct) or with different concentrations of NSC23766 (NSC, μM) for 30 min (n = 2306, 2019, 1845 or 1282 sperm for Ct, NSC5, NSC25 or NSC50, respectively) and 60 min (n = 1730, 1673, 1131 or 528 sperm for Ct, NSC5, NSC25 or NSC50, respectively). For each condition, the percentage of motile sperm (mean ± standard error, n = 7 samples) is also shown (bottom boxes). Statistically significant differences between treatments are indicated by distinct letters (*Ps* < 0.001).
(TIF)

S5 Fig Effect of RAC1 inhibition by EHop-016 on progressive motility of sperm from *t* heterozygous (*t*/+) mice. Histograms showing the frequency distribution of sperm over linearity (LIN; %) bins in *t*/+ samples (n = 4) incubated without (controls; upper graph; n = 1097 sperm) or with (lower graph; n = 1095 sperm) 2.5 μM EHop-016 for 30 min.
(TIF)

S6 Fig Molecular model of transmission ratio distortion comprising the role of RAC1. Schematic drawing representing two haploid spermatids connected by a cytoplasmic bridge allowing the exchange of RNA and proteins (horizontal arrows), expressing the wild type (+; genes and gene products in green) or *t*-haplotype (*t*; genes and gene products in dark red) variant of chromosome 17. Distorter gene products from either genotype act in both cells on Rho GTPase pathways effecting activation (through factor Y) or inhibition (through factor X) of SMOK, which controls sperm progressive motility. Upregulation of the activating pathway and parallel downregulation of the inhibitory pathway impair progressive motility in +-sperm, while t-sperm is protected by dominant-negative SMOK^TCR^, which is retained in and thus exclusively rescues progressive motility of *t*-sperm. Lower panel: The RAC1 inhibitor NSC23766 attenuates the effect of enhanced RAC1 activity caused by TIAM2S in both cells and thereby rescues progressive motility in +-sperm (in a strictly dosage dependent manner). Thus, it adopts the role of SMOK^TCR^ missing in +-sperm. SMOK activity in *t*-sperm and +-sperm are approximated by NSC23766 (5 μM) treatment, therefore *t*-sperm and +-sperm are equalized with respect to progressive motility. RAC1 in the repressive pathway might be low due to down-regulation of the *t*-allele of TIAM2L, and thus, inhibition by NSC23766 might not significantly reduce the activity of factor X, which is also controlled by TAGAP [16]. Arrows indicate activation, blocked bars inhibition. Red upward pointing arrows at distorter proteins indicate up-regulation, blue down-pointing arrows down-regulation of the *t* variant; the green down-pointing arrow to the axoneme symbolizes normal or rescued, the dark-red down-pointing arrow impaired progressive motility. Grey gene symbols, X and Y indicate postulated factors.
(PDF)

S1 Video Representative video of a wild-type (+/+) sample captured after 30 min incubation without NSC23766 (control). All videos show mouse sperm tracks captured by CASA. Tracks color code is as following: blue – highly progressive motile sperm; green – motile, but less progressive sperm; red – immotile sperm; yellow – late tracks (recording started after the first 10 frames; excluded from analysis).
(MP4)

S2 Video Representative video of a wild-type (+/+) sample captured after 30 min incubation with 5 μM NSC. (MP4)

S3 Video Representative video of a wild-type (+/+) sample captured after 30 min incubation with 25 μM NSC. (MP4)

S4 Video Representative video of a wild-type (+/+) sample captured after 30 min incubation with 50 μM NSC. (MP4)

S5 Video Representative video of a sperm sample isolated from a wild type (+/+) mouse. (MP4)

S6 Video Representative video of a sperm sample isolated from a heterozygous *t* (*t*/+) mouse. (MP4)

S7 Video Representative video of a sperm sample isolated from a homozygous *t* (*t*/*t*) mouse. (MP4)

S8 Video Representative video of a heterozygous *t* (*t*/+) sample captured after 30 min incubation without NSC23766. (MP4)

S9 Video Representative video of a heterozygous *t* (*t*/+) sample captured after 30 min incubation with 5 μM NSC23766. (MP4)

S10 Video Representative video of a heterozygous *t* (*t/+*) sample captured immediately after sperm isolation and processing. For training purposes tracks were omitted. Three highly progressive or less progressive sperm each are indicated.
(PPTX)

S1 Dataset NSC23766 treatment of wild type (+/+) mouse sperm. S1 to S8 datasets comprise the values of individual motility parameters of each sperm analyzed. Each 7-column line (VAP, VSL, VCL, ALH, BCF, STR and LIN) represents one sperm track.
(XLSX)

S2 Dataset Percentage of motile sperm in each mouse or bull sample analyzed. (XLSX)

S3 Dataset EHop-016 treatment of wild type (+/+) mouse sperm. (XLSX)

S4 Dataset Sperm from males of different *t* genotypes. (XLSX)

S5 Dataset NSC23766 treatment of heterozygous *t* (*t*/+) mouse sperm. (XLSX)

S6 Dataset EHop-016 treatment of heterozygous *t* (*t*/+) mouse sperm. (XLSX)

S7 Dataset NSC23766 treatment of homozygous *t* (*t*/*t*) mouse sperm. (XLSX)

S8 Dataset NSC23766 treatment of bull sperm. (XLSX)

S9 Dataset EHop-016 treatment of bull sperm. (XLSX)

## References

[1] Turner RM. Tales from the tail: what do we really know about sperm motility? J Androl. 2003; 24(6):790–803. 10.1002/j.1939-4640.2003.tb03123.x . 14581499

[2] Turner RM. Moving to the beat: a review of mammalian sperm motility regulation. Reprod Fertil Dev. 2006; 18(1–2):25–38. 10.1071/rd05120 . 16478600

[3] Kaupp UB, Strunker T. Signaling in Sperm: More Different than Similar. Trends Cell Biol. 2017; 27(2):101–9. 10.1016/j.tcb.2016.10.002 . 27825709

[4] Pereira R, Sa R, Barros A, Sousa M. Major regulatory mechanisms involved in sperm motility. Asian J Androl. 2017; 19(1):5–14. 10.4103/1008-682X.167716 26680031 PMC5227674

[5] Lindemann CB, Lesich KA. Flagellar and ciliary beating: the proven and the possible. J Cell Sci. 2010; 123(Pt 4):519–28. 10.1242/jcs.051326 . 20145000

[6] Inaba K. Sperm flagella: comparative and phylogenetic perspectives of protein components. Mol Hum Reprod. 2011; 17(8):524–38. 10.1093/molehr/gar034 . 21586547

[7] Viswanadha R, Sale WS, Porter ME. Ciliary Motility: Regulation of Axonemal Dynein Motors. Cold Spring Harb Perspect Biol. 2017; 9(8). 10.1101/cshperspect.a018325 28765157 PMC5538414

[8] Inaba K, Shiba K. Microscopic analysis of sperm movement: links to mechanisms and protein components. Microscopy (Oxf). 2018; 67(3):144–55. 10.1093/jmicro/dfy021 . 29741637

[9] Schimenti J. Segregation distortion of mouse t haplotypes the molecular basis emerges. Trends Genet. 2000; 16(6):240–3. 10.1016/s0168-9525(00)02020-5 . 10827448

[10] Herrmann BG, Bauer H. The mouse t-haplotype: a selfish chromosome—genetics, molecular mechanism, and evolution. Evolution of the House Mouse. Cambridge, UK: Cambridge University Press; 2012 p. 297–314.

[11] Herrmann BG, Koschorz B, Wertz K, McLaughlin KJ, Kispert A. A protein kinase encoded by the t complex responder gene causes non-mendelian inheritance. Nature. 1999; 402(6758):141–6. 10.1038/45970 . 10647005

[12] Lyon MF. Transmission ratio distortion in mice. Annu Rev Genet. 2003; 37:393–408. 10.1146/annurev.genet.37.110801.143030 . 14616067

[13] Bauer H, Willert J, Koschorz B, Herrmann BG. The t complex-encoded GTPase-activating protein Tagap1 acts as a transmission ratio distorter in mice. Nat Genet. 2005; 37(9):969–73. 10.1038/ng1617 . 16116428

[14] Bauer H, Veron N, Willert J, Herrmann BG. The t-complex-encoded guanine nucleotide exchange factor Fgd2 reveals that two opposing signaling pathways promote transmission ratio distortion in the mouse. Genes Dev. 2007; 21(2):143–7. 10.1101/gad.414807 17234881 PMC1770897

[15] Bauer H, Schindler S, Charron Y, Willert J, Kusecek B, Herrmann BG. The nucleoside diphosphate kinase gene Nme3 acts as quantitative trait locus promoting non-Mendelian inheritance. PLoS Genet. 2012; 8(3):e1002567 10.1371/journal.pgen.1002567 22438820 PMC3305403

[16] Charron Y, Willert J, Lipkowitz B, Kusecek B, Herrmann BG, Bauer H. Two isoforms of the RAC-specific guanine nucleotide exchange factor TIAM2 act oppositely on transmission ratio distortion by the mouse t-haplotype. PLoS Genet. 2019; 15(2):e1007964 10.1371/journal.pgen.1007964 30817801 PMC6394906

[17] Olds-Clarke P. Motility characteristics of sperm from the uterus and oviducts of female mice after mating to congenic males differing in sperm transport and fertility. Biol Reprod. 1986; 34(3):453–67. 10.1095/biolreprod34.3.453 . 3697462

[18] Olds-Clarke P, Johnson LR. t haplotypes in the mouse compromise sperm flagellar function. Dev Biol. 1993; 155(1):14–25. 10.1006/dbio.1993.1002 . 8416830

[19] Kuretake S, Maleszewski M, Tokumasu A, Fujimoto H, Yanagimachi R. Inadequate function of sterile tw5/tw32 spermatozoa overcome by intracytoplasmic sperm injection. Mol Reprod Dev. 1996; 44(2):230–3. 10.1002/(SICI)1098-2795(199606)44:2&lt;230::AID-MRD12&gt;3.0.CO;2-6 . 9115721

[20] Tessler S, Carey JE, Olds-Clarke P. Mouse sperm motility affected by factors in the T/t complex. J Exp Zool. 1981; 217(2):277–85. 10.1002/jez.1402170214 . 7288391

[21] Olds-Clarke P. The nonprogressive motility of sperm populations from mice with a tw32 haplotype. J Androl. 1983; 4(2):136–43. 10.1002/j.1939-4640.1983.tb00738.x . 6853358

[22] Katz DF, Erickson RP, Nathanson M. Beat frequency is bimodally distributed in spermatozoa from T/t12 mice. J Exp Zool. 1979; 210(3):529–35. 10.1002/jez.1402100316 . 541605

[23] Tessler S, Olds-Clarke P. Male genotype influences sperm transport in female mice. Biol Reprod. 1981; 24(4):806–13. 10.1095/biolreprod24.4.806 . 7248414

[24] Hall A. Rho family GTPases. Biochem Soc Trans. 2012; 40(6):1378–82. 10.1042/BST20120103 . 23176484

[25] Hanna S, El-Sibai M. Signaling networks of Rho GTPases in cell motility. Cell Signal. 2013; 25(10):1955–61. 10.1016/j.cellsig.2013.04.009 . 23669310

[26] Devreotes P, Horwitz AR. Signaling networks that regulate cell migration. Cold Spring Harb Perspect Biol. 2015; 7(8):a005959 10.1101/cshperspect.a005959 26238352 PMC4526752

[27] Biro M, Munoz MA, Weninger W. Targeting Rho-GTPases in immune cell migration and inflammation. Br J Pharmacol. 2014; 171(24):5491–506. 10.1111/bph.12658 24571448 PMC4282076

[28] Van Haastert PJ, Devreotes PN. Chemotaxis: signalling the way forward. Nat Rev Mol Cell Biol. 2004; 5(8):626–34. Epub 2004/09/16. 10.1038/nrm1435 . 15366706

[29] Cherfils J, Zeghouf M. Regulation of small GTPases by GEFs, GAPs, and GDIs. Physiol Rev. 2013; 93(1):269–309. 10.1152/physrev.00003.2012 . 23303910

[30] Veron N, Bauer H, Weisse AY, Luder G, Werber M, Herrmann BG. Retention of gene products in syncytial spermatids promotes non-Mendelian inheritance as revealed by the t complex responder. Genes Dev. 2009; 23(23):2705–10. 10.1101/gad.553009 19952105 PMC2788329

[31] Giojalas LC, Rovasio RA. Mouse spermatozoa modify their motility parameters and chemotactic response to factors from the oocyte microenvironment. Int J Androl. 1998; 21(4):201–6. . 9749350

[32] de Beco S, Vaidziulyte K, Manzi J, Dalier F, di Federico F, Cornilleau G, et al Optogenetic dissection of Rac1 and Cdc42 gradient shaping. Nat Commun. 2018; 9(1):4816 Epub 2018/11/18. 10.1038/s41467-018-07286-8 30446664 PMC6240110

[33] Wachten D, Jikeli JF, Kaupp UB. Sperm Sensory Signaling. Cold Spring Harb Perspect Biol. 2017; 9(7). 10.1101/cshperspect.a028225 28062561 PMC5495058

[34] Balbach M, Beckert V, Hansen JN, Wachten D. Shedding light on the role of cAMP in mammalian sperm physiology. Mol Cell Endocrinol. 2018; 468:111–20. 10.1016/j.mce.2017.11.008 . 29146556

[35] Pankov R, Endo Y, Even-Ram S, Araki M, Clark K, Cukierman E, et al A Rac switch regulates random versus directionally persistent cell migration. J Cell Biol. 2005; 170(5):793–802. 10.1083/jcb.200503152 16129786 PMC2171343

[36] Marei H, Malliri A. GEFs: Dual regulation of Rac1 signaling. Small GTPases. 2017; 8(2):90–9. 10.1080/21541248.2016.1202635 27314616 PMC5464116

[37] Di Ciano-Oliveira C, Thirone AC, Szaszi K, Kapus A. Osmotic stress and the cytoskeleton: the R(h)ole of Rho GTPases. Acta Physiol (Oxf). 2006; 187(1–2):257–72. 10.1111/j.1748-1716.2006.01535.x . 16734763

[38] Ortega MC, Santander-Garcia D, Marcos-Ramiro B, Barroso S, Cox S, Jimenez-Alfaro I, et al Activation of Rac1 and RhoA Preserve Corneal Endothelial Barrier Function. Invest Ophthalmol Vis Sci. 2016; 57(14):6210–22. 10.1167/iovs.16-20031 . 27849309

[39] Rawe VY, Ramalho-Santos J, Payne C, Chemes HE, Schatten G. WAVE1, an A-kinase anchoring protein, during mammalian spermatogenesis. Hum Reprod. 2004; 19(11):2594–604. 10.1093/humrep/deh513 . 15471936

[40] Ducummon CC, Berger T. Localization of the Rho GTPases and some Rho effector proteins in the sperm of several mammalian species. Zygote. 2006; 14(3):249–57. 10.1017/S0967199406003790 . 16822336

[41] Delgado-Buenrostro NL, Mujica A, Chiquete-Felix N, Deciga-Alcaraz A, Medina-Reyes EI, Uribe-Carvajal S, et al Role of Wasp and the small GTPases RhoA, RhoB, and Cdc42 during capacitation and acrosome reaction in spermatozoa of English guinea pigs. Mol Reprod Dev. 2016; 83(10):927–37. 10.1002/mrd.22657 . 27182927

[42] Delgado-Buenrostro NL, Hernandez-Gonzalez EO, Segura-Nieto M, Mujica A. Actin polymerization in the equatorial and postacrosomal regions of guinea pig spermatozoa during the acrosome reaction is regulated by G proteins. Mol Reprod Dev. 2005; 70(2):198–210. 10.1002/mrd.20192 . 15570614

[43] Fiedler SE, Bajpai M, Carr DW. Identification and characterization of RHOA-interacting proteins in bovine spermatozoa. Biol Reprod. 2008; 78(1):184–92. 10.1095/biolreprod.107.062943 . 17928627

[44] Baltierrez-Hoyos R, Roa-Espitia AL, Hernandez-Gonzalez EO. The association between CDC42 and caveolin-1 is involved in the regulation of capacitation and acrosome reaction of guinea pig and mouse sperm. Reproduction. 2012; 144(1):123–34. 10.1530/REP-11-0433 . 22596063

[45] Romarowski A, Battistone MA, La Spina FA, Puga Molina Ldel C, Luque GM, Vitale AM, et al PKA-dependent phosphorylation of LIMK1 and Cofilin is essential for mouse sperm acrosomal exocytosis. Dev Biol. 2015; 405(2):237–49. 10.1016/j.ydbio.2015.07.008 26169470 PMC4546557

[46] Angeles-Floriano T, Roa-Espitia AL, Baltierrez-Hoyos R, Cordero-Martinez J, Elizondo G, Hernandez-Gonzalez EO. Absence of aryl hydrocarbon receptor alters CDC42 expression and prevents actin polymerization during capacitation. Mol Reprod Dev. 2016; 83(11):1015–26. 10.1002/mrd.22736 . 27635527

[47] Ramirez-Ramirez D, Salgado-Lucio ML, Roa-Espitia AL, Fierro R, Gonzalez-Marquez H, Cordero-Martinez J, et al Rac1 is necessary for capacitation and acrosome reaction in guinea pig spermatozoa. J Cell Biochem. 2020; 121(4):2864–76. 10.1002/jcb.29521 WOS:000494566500001. 31692044

[48] Hinsch KD, Habermann B, Just I, Hinsch E, Pfisterer S, Schill WB, et al ADP-ribosylation of Rho proteins inhibits sperm motility. FEBS Lett. 1993; 334(1):32–6. 10.1016/0014-5793(93)81674-o . 8224222

[49] Nakamura K, Fujita A, Murata T, Watanabe G, Mori C, Fujita J, et al Rhophilin, a small GTPase Rho-binding protein, is abundantly expressed in the mouse testis and localized in the principal piece of the sperm tail. FEBS Lett. 1999; 445(1):9–13. 10.1016/s0014-5793(99)00087-3 . 10069364

[50] Fujita A, Nakamura K, Kato T, Watanabe N, Ishizaki T, Kimura K, et al Ropporin, a sperm-specific binding protein of rhophilin, that is localized in the fibrous sheath of sperm flagella. J Cell Sci. 2000; 113 (Pt 1):103–12. . 10591629 10.1242/jcs.113.1.103

[51] Fiedler SE, Dudiki T, Vijayaraghavan S, Carr DW. Loss of R2D2 proteins ROPN1 and ROPN1L causes defects in murine sperm motility, phosphorylation, and fibrous sheath integrity. Biol Reprod. 2013; 88(2):41 10.1095/biolreprod.112.105262 23303679 PMC4434999

[52] Parte PP, Rao P, Redij S, Lobo V, D'Souza SJ, Gajbhiye R, et al Sperm phosphoproteome profiling by ultra performance liquid chromatography followed by data independent analysis (LC-MS(E)) reveals altered proteomic signatures in asthenozoospermia. J Proteomics. 2012; 75(18):5861–71. 10.1016/j.jprot.2012.07.003 . 22796355

[53] Amaral A, Paiva C, Attardo Parrinello C, Estanyol JM, Ballesca JL, Ramalho-Santos J, et al Identification of proteins involved in human sperm motility using high-throughput differential proteomics. J Proteome Res. 2014; 13(12):5670–84. 10.1021/pr500652y . 25250979

[54] Wittmann T, Waterman-Storer CM. Cell motility: can Rho GTPases and microtubules point the way? J Cell Sci. 2001; 114(Pt 21):3795–803. . 11719546 10.1242/jcs.114.21.3795

[55] Pleines I, Dutting S, Cherpokova D, Eckly A, Meyer I, Morowski M, et al Defective tubulin organization and proplatelet formation in murine megakaryocytes lacking Rac1 and Cdc42. Blood. 2013; 122(18):3178–87. 10.1182/blood-2013-03-487942 . 23861250

[56] Song H, Bush RA, Vijayasarathy C, Fariss RN, Kjellstrom S, Sieving PA. Transgenic expression of constitutively active RAC1 disrupts mouse rod morphogenesis. Invest Ophthalmol Vis Sci. 2014; 55(4):2659–68. 10.1167/iovs.13-13649 24651551 PMC4001786

[57] Wojnacki J, Quassollo G, Marzolo MP, Caceres A. Rho GTPases at the crossroad of signaling networks in mammals: impact of Rho-GTPases on microtubule organization and dynamics. Small GTPases. 2014; 5:e28430 10.4161/sgtp.28430 24691223 PMC4114925

[58] Gervasi MG, Xu X, Carbajal-Gonzalez B, Buffone MG, Visconti PE, Krapf D. The actin cytoskeleton of the mouse sperm flagellum is organized in a helical structure. J Cell Sci. 2018; 131(11). 10.1242/jcs.215897 29739876 PMC6031324

[59] Azamar Y, Uribe S, Mujica A. F-actin involvement in guinea pig sperm motility. Mol Reprod Dev. 2007; 74(3):312–20. 10.1002/mrd.20578 . 16998842

[60] Liu DY, Martic M, Clarke GN, Dunlop ME, Baker HW. An important role of actin polymerization in the human zona pellucida-induced acrosome reaction. Mol Hum Reprod. 1999; 5(10):941–9. 10.1093/molehr/5.10.941 . 10508222

[61] Finkelstein M, Megnagi B, Ickowicz D, Breitbart H. Regulation of sperm motility by PIP2(4,5) and actin polymerization. Dev Biol. 2013; 381(1):62–72. 10.1016/j.ydbio.2013.06.014 . 23791551

[62] Correa LM, Thomas A, Meyers SA. The macaque sperm actin cytoskeleton reorganizes in response to osmotic stress and contributes to morphological defects and decreased motility. Biol Reprod. 2007; 77(6):942–53. 10.1095/biolreprod.107.060533 . 17823088

[63] Lobo V, Parte P. Membrane-bound Glucose regulated protein 78 interacts with alpha-2-macroglobulin to promote actin reorganization in sperm during epididymal maturation. Mol Hum Reprod. 2019; 25(3):137–55. 10.1093/molehr/gay055 . 30590815

[64] Gao Y, Xing J, Streuli M, Leto TL, Zheng Y. Trp(56) of rac1 specifies interaction with a subset of guanine nucleotide exchange factors. J Biol Chem. 2001; 276(50):47530–41. 10.1074/jbc.M108865200 . 11595749

[65] Gao Y, Dickerson JB, Guo F, Zheng J, Zheng Y. Rational design and characterization of a Rac GTPase-specific small molecule inhibitor. Proc Natl Acad Sci U S A. 2004; 101(20):7618–23. 10.1073/pnas.0307512101 15128949 PMC419655

[66] Montalvo-Ortiz BL, Castillo-Pichardo L, Hernandez E, Humphries-Bickley T, De la Mota-Peynado A, Cubano LA, et al Characterization of EHop-016, novel small molecule inhibitor of Rac GTPase. J Biol Chem. 2012; 287(16):13228–38. 10.1074/jbc.M111.334524 22383527 PMC3339933

[67] Amann RP, Waberski D. Computer-assisted sperm analysis (CASA): capabilities and potential developments. Theriogenology. 2014; 81(1):5–17 e1-3. 10.1016/j.theriogenology.2013.09.004 . 24274405

[68] Mortimer ST, van der Horst G, Mortimer D. The future of computer-aided sperm analysis. Asian J Androl. 2015; 17(4):545–53. 10.4103/1008-682X.154312 25926614 PMC4492043

