# Supplementary figures and images for: RAC1 controls progressive movement and competitiveness of mammalian spermatozoa
Source: PLoS Genet. 2021 Feb 4;17(2):e1009308. doi: 10.1371/journal.pgen.1009308 (PMC7861394; doi:10.1371/journal.pgen.1009308)

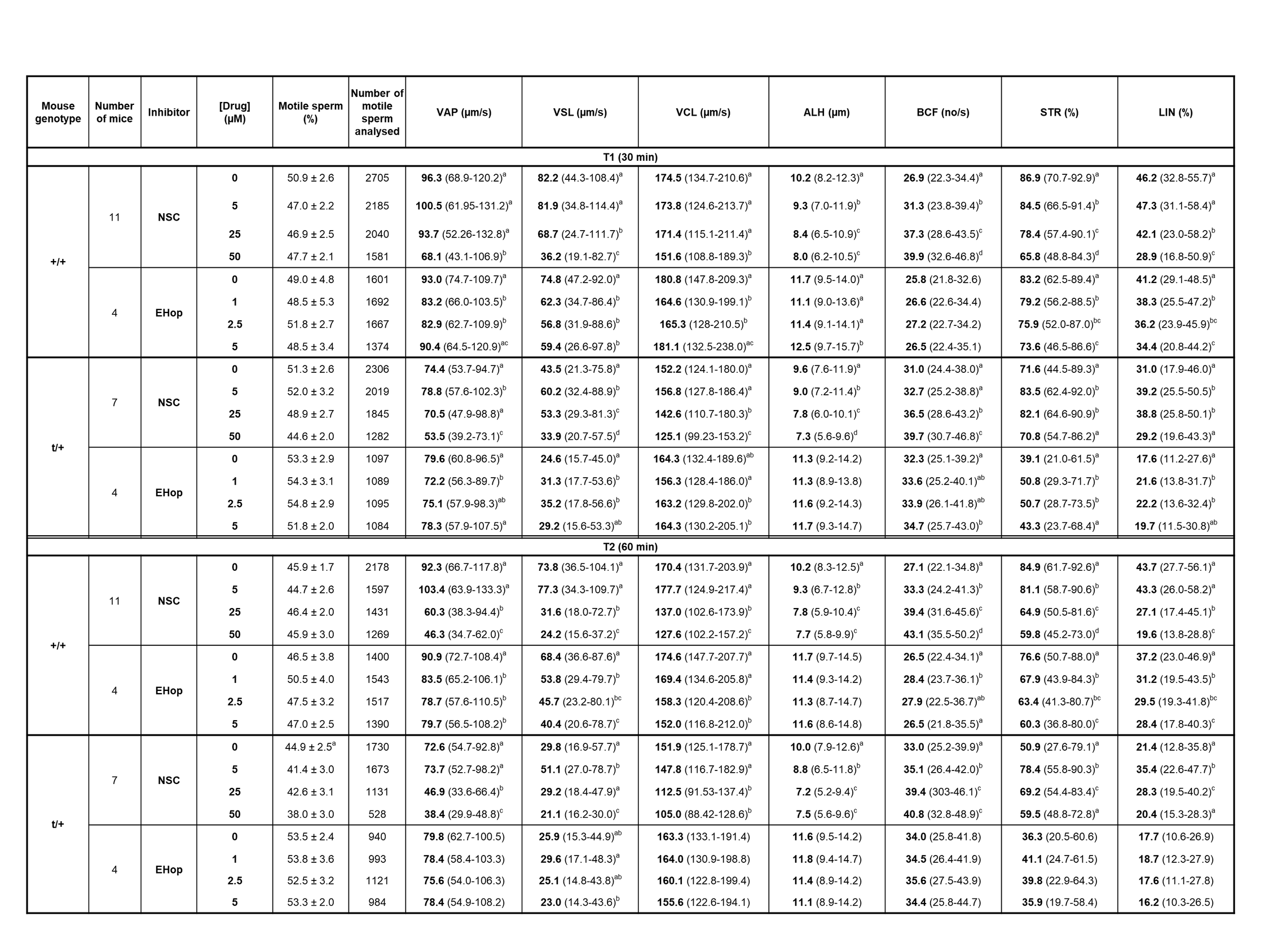

Supplement: S1 Table — The percentage of motile sperm is given as the mean ± standard error. Kinematic parameters are expressed as median (with 25th-75th percentiles). For each parameter, within each genotype, statistically significant differences between treatments are indicated by different letters (Ps < 0.001). (TIF) [file pgen.1009308.s001.tif]

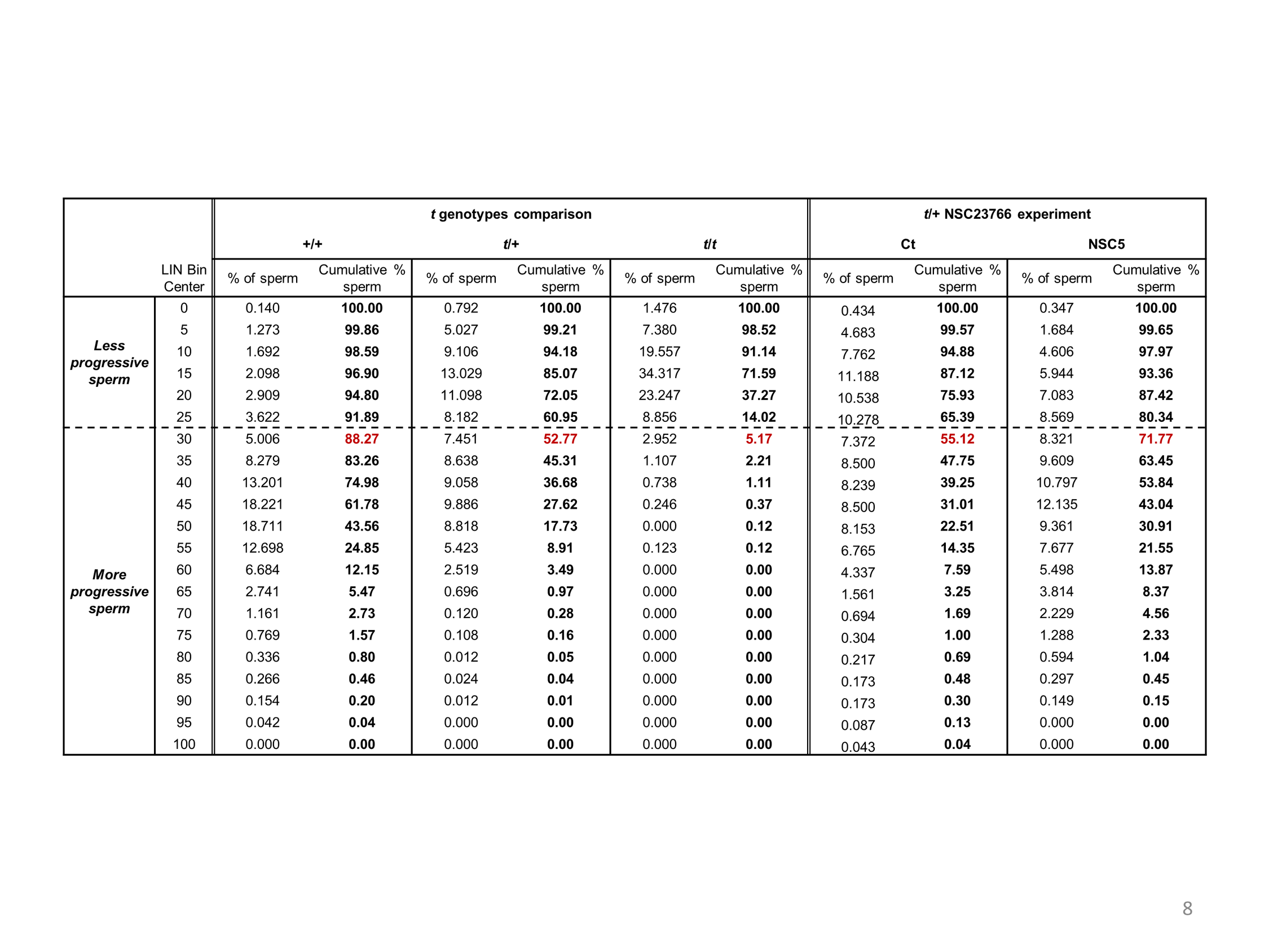

Supplement: S2 Table — Percentages of sperm in each linearity (LIN) BIN and cumulative percentages of more progressive sperm (sum indicated in red; dashed line indicates the transition between the +/+ and t/t profile) are shown. (Left side) Comparison between sperm from wild type (+/+; n = 7151), heterozygous t (t/+; n = 8335) and homozygous t (t/t; n = 813) mice. (Right side) Comparison between sperm from t/+ samples incubated without (control–Ct; n = 2306 sperm) or with 5 μM NSC23766 (NSC5; n = 2019 sperm) for 30 min. (TIF) [file pgen.1009308.s002.tif]

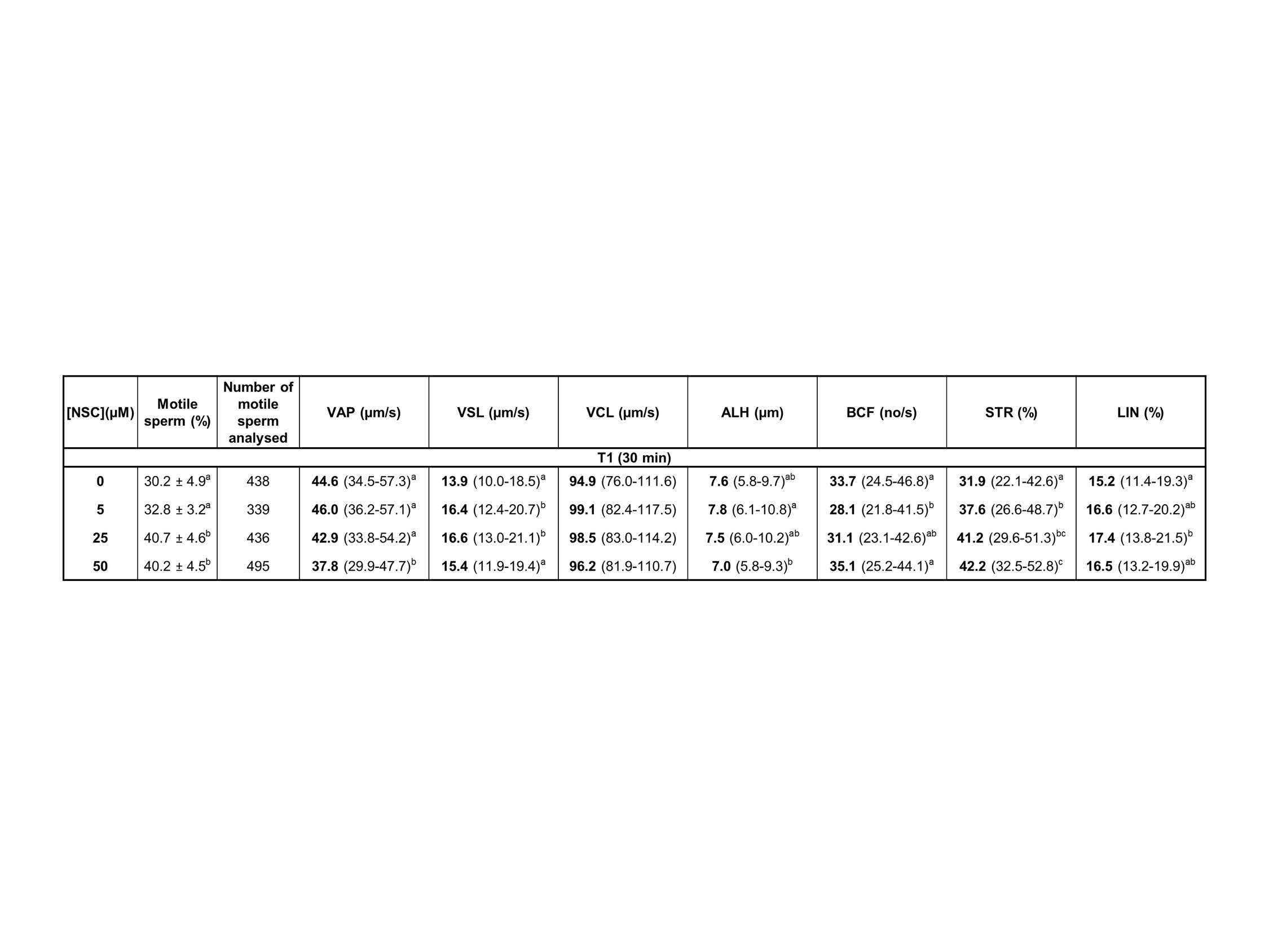

Supplement: S3 Table — The percentage of motile sperm is given as the mean ± standard error. Kinematic parameters are expressed as median (with 25th-75th percentiles). Statistically significant differences between treatments are indicated by different letters (Ps < 0.01). (TIF) [file pgen.1009308.s003.tif]

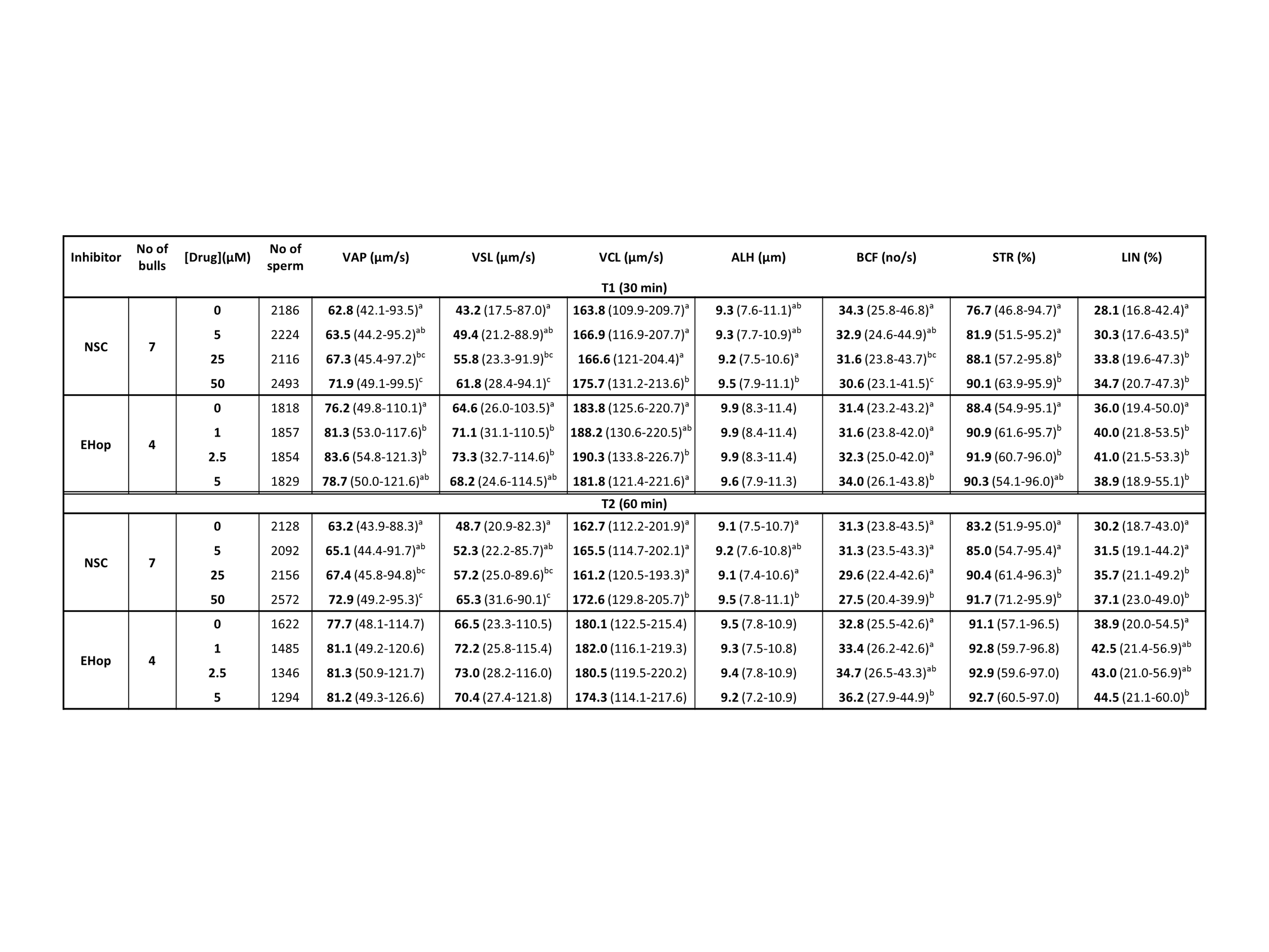

Supplement: S4 Table — The percentage of motile sperm is given as the mean ± standard error. Kinematic parameters are expressed as median (with 25th-75th percentiles). Statistically significant differences between treatments are indicated by distinct letters (Ps < 0.001). (TIF) [file pgen.1009308.s004.tif]

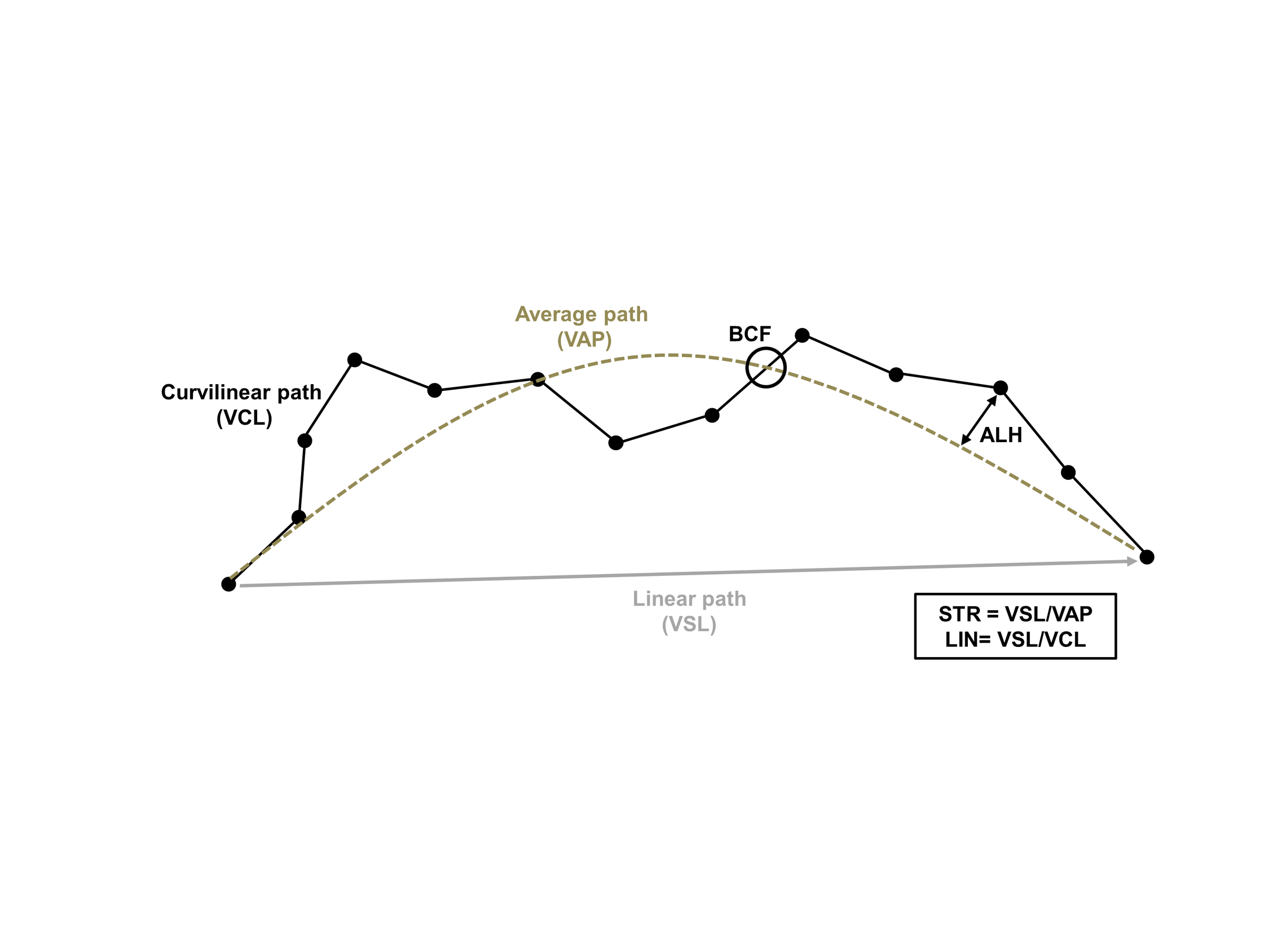

Supplement: S1 Fig — Schematic drawing explaining the kinematic parameters computed by CASA. Three trajectories are established: curvilinear path (real trajectory), average path, and linear path (straight line trajectory). Three velocities (μm/s) are calculated based on these paths: curvilinear velocity (VCL), average path velocity (VAP) and straight-line velocity (VSL). The amplitude of the lateral head displacement (ALH; μm) is the deviation of a sperm head from its average path. Beat cross frequency (BCF; number per second) is the average rate at which the curvilinear path crosses the average path (a derivation of the frequencies of rotation of the head and of flagellar beat). The linearity of the average path is given by straightness (STR = VSL/VAP; %) and the linearity of the curvilinear path is called linearity (LIN = VSL/VCL; %). (TIF) [file pgen.1009308.s005.tif]

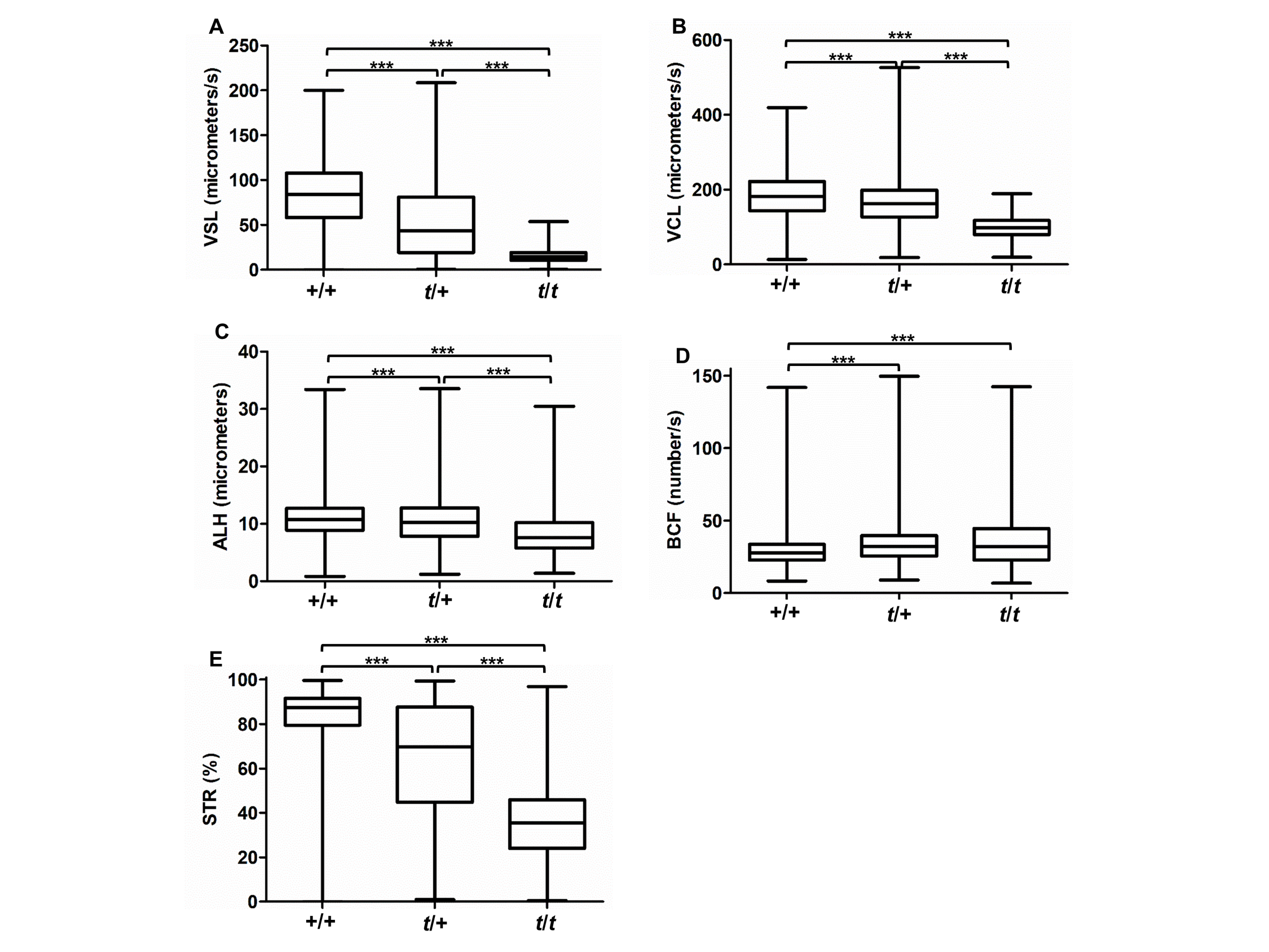

Supplement: S2 Fig — Box plots (showing the median and first and third quartiles) with whiskers (from minimum to maximum) of straight-line velocity (VSL; A), curvilinear velocity (VCL; B), amplitude of the lateral head displacement (ALH; C), beat cross frequency (BCF; D) and straightness (STR; E) of sperm from wild type (+/+; n = 7151 sperm), heterozygous t (t/+; n = 8835 sperm) and homozygous t (t/t; n = 813 sperm) mice (n = 15 mice for each genotype), immediately after sperm isolation. Asterisks indicate statistically significant differences between genotypes (***Ps < 0.001). (TIF) [file pgen.1009308.s006.tif]

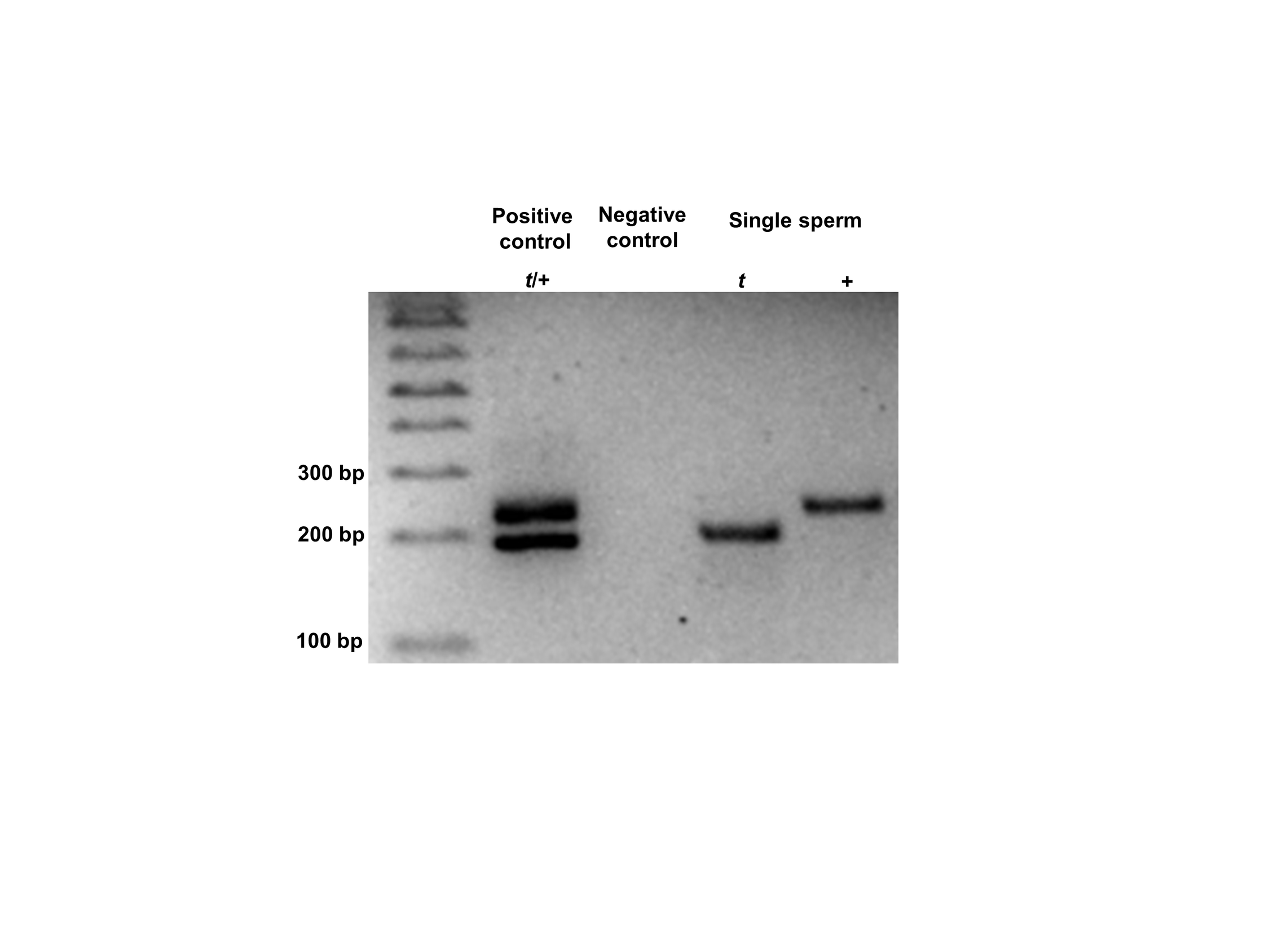

Supplement: S3 Fig — Gel image of DNA fragments amplified by PCR of t/+, single +-sperm or t-sperm (isolated by micro-pipetting of highly or less progressive sperm), and separated by agarose gel electrophoresis (+ band: 228 bp; t band: 195 bp). t/+ sperm and cell-free culture medium served as positive and negative controls, respectively. (TIF) [file pgen.1009308.s007.tif]

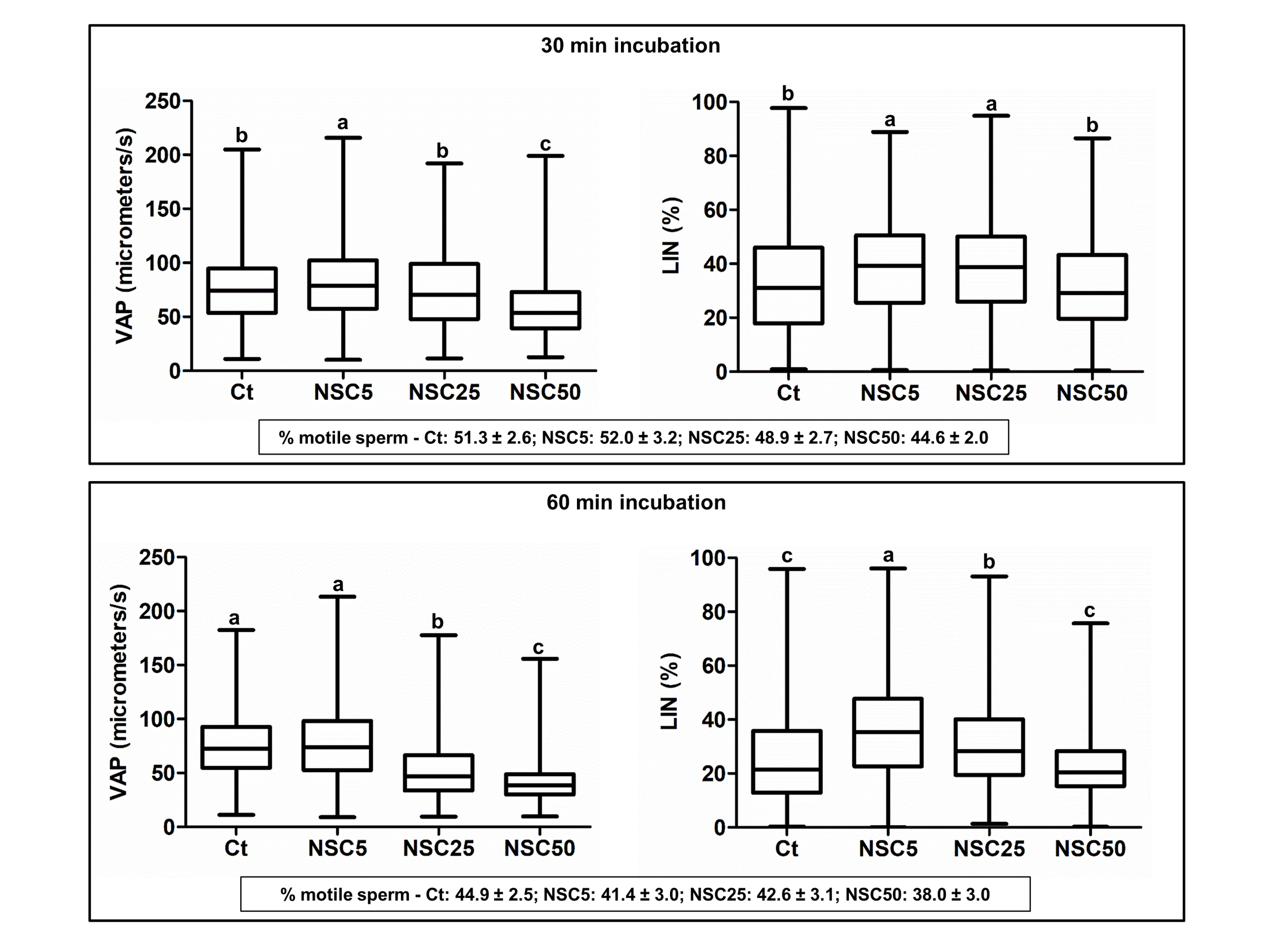

Supplement: S4 Fig — Box plots (showing the median and first and third quartiles) with whiskers (from minimum to maximum) of average path velocity (VAP) and linearity (LIN) of sperm incubated without (controls–Ct) or with different concentrations of NSC23766 (NSC, μM) for 30 min (n = 2306, 2019, 1845 or 1282 sperm for Ct, NSC5, NSC25 or NSC50, respectively) and 60 min (n = 1730, 1673, 1131 or 528 sperm for Ct, NSC5, NSC25 or NSC50, respectively). For each condition, the percentage of motile sperm (mean ± standard error, n = 7 samples) is also shown (bottom boxes). Statistically significant differences between treatments are indicated by distinct letters (Ps < 0.001). (TIF) [file pgen.1009308.s008.tif]

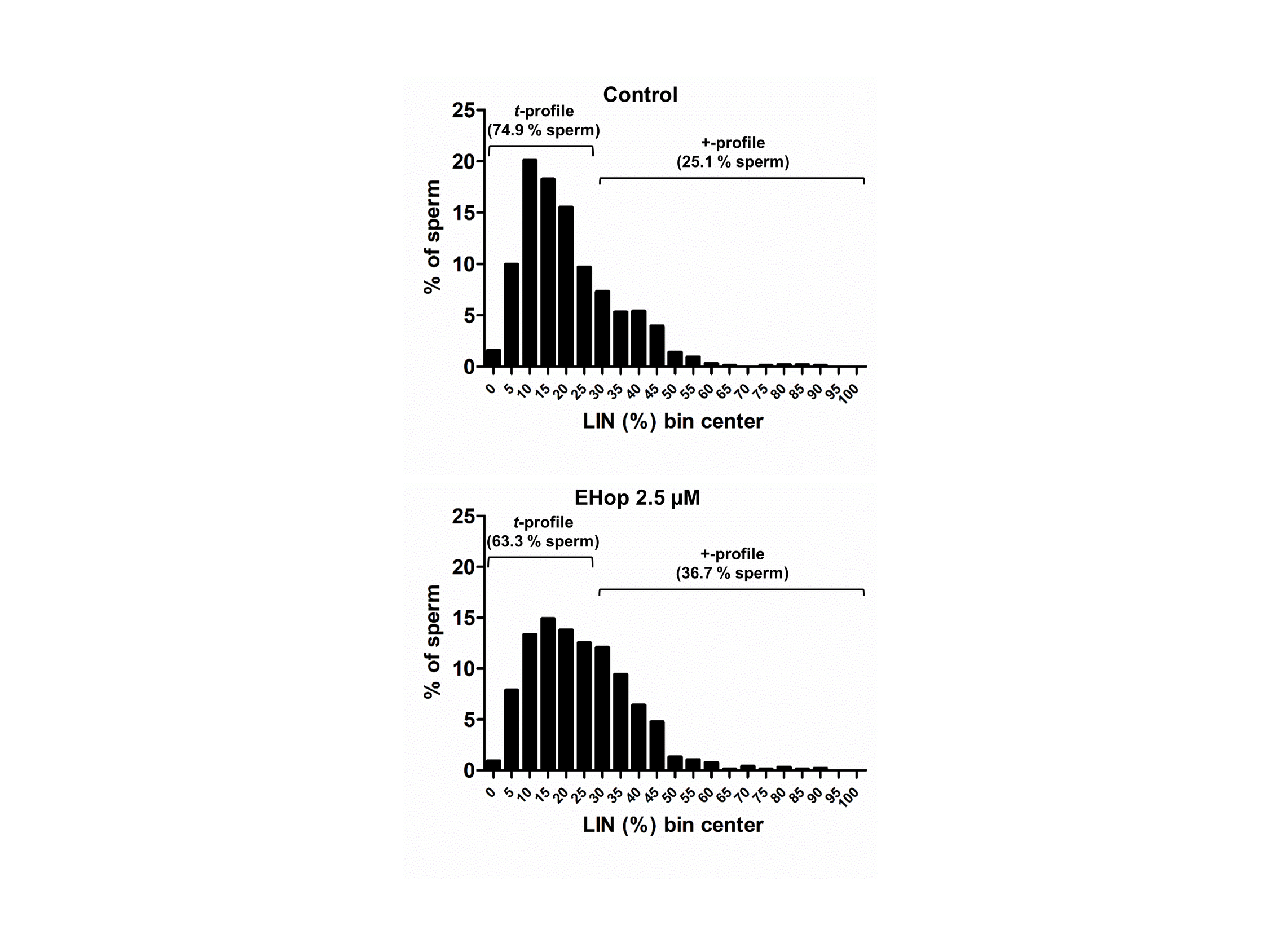

Supplement: S5 Fig — Histograms showing the frequency distribution of sperm over linearity (LIN; %) bins in t/+ samples (n = 4) incubated without (controls; upper graph; n = 1097 sperm) or with (lower graph; n = 1095 sperm) 2.5 μM EHop-016 for 30 min. (TIF) [file pgen.1009308.s009.tif]

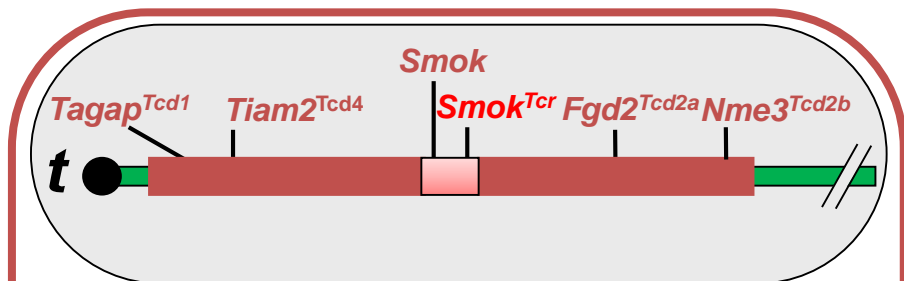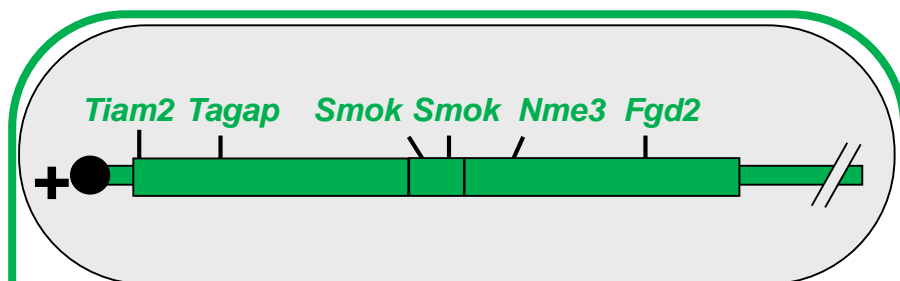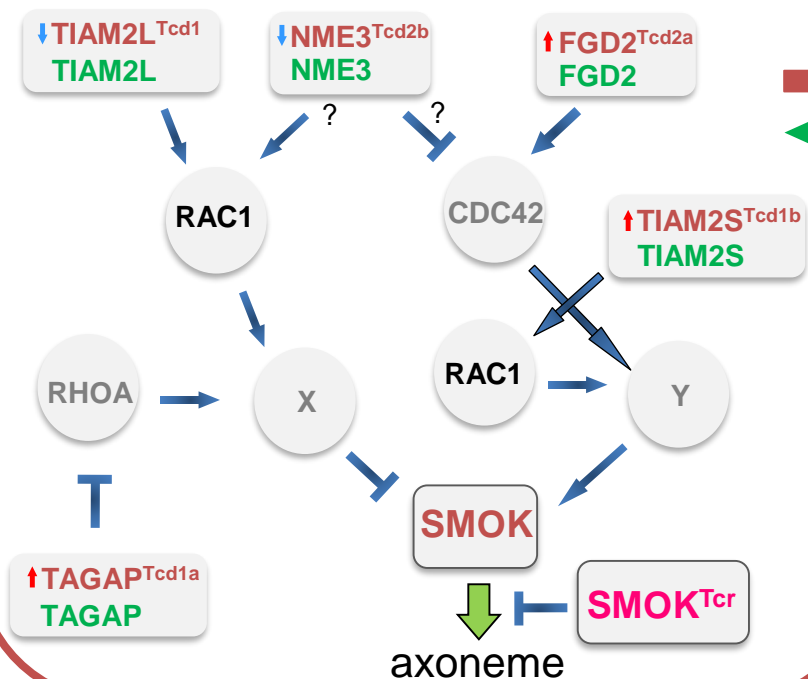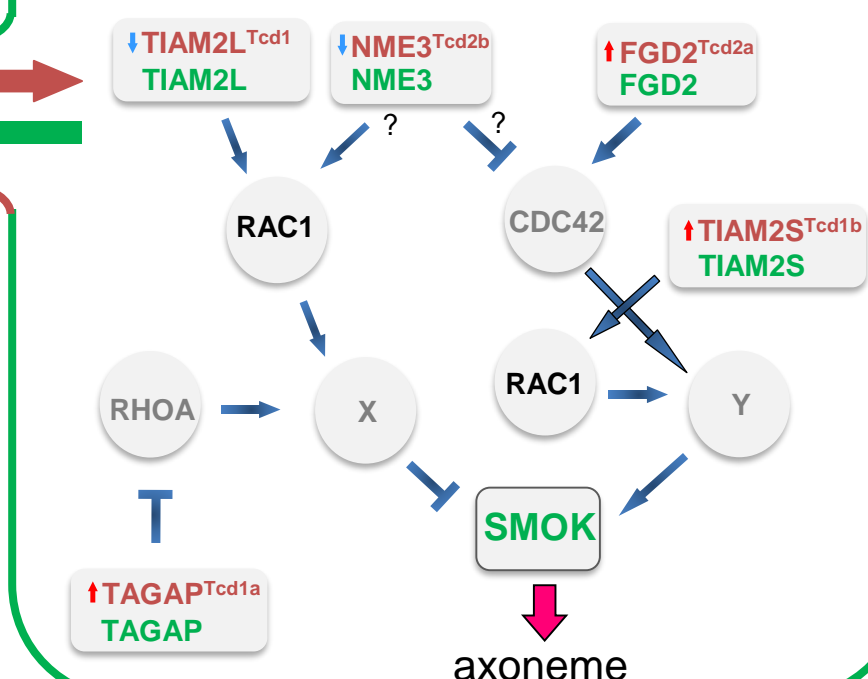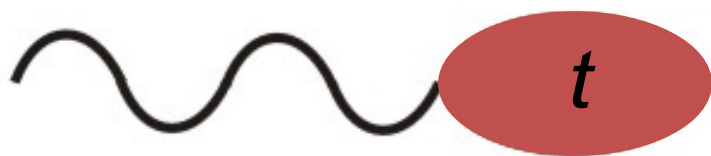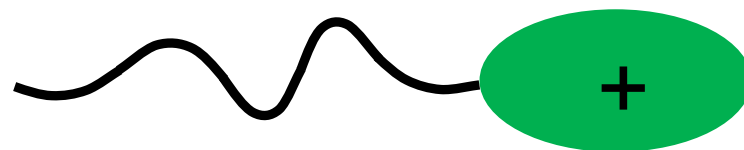

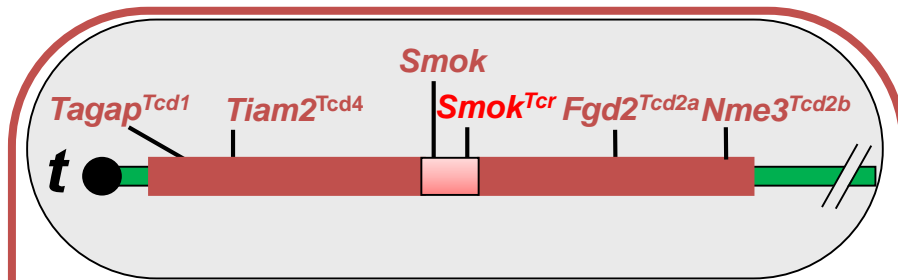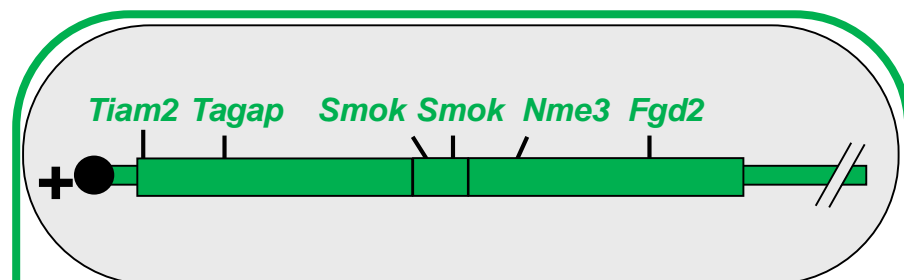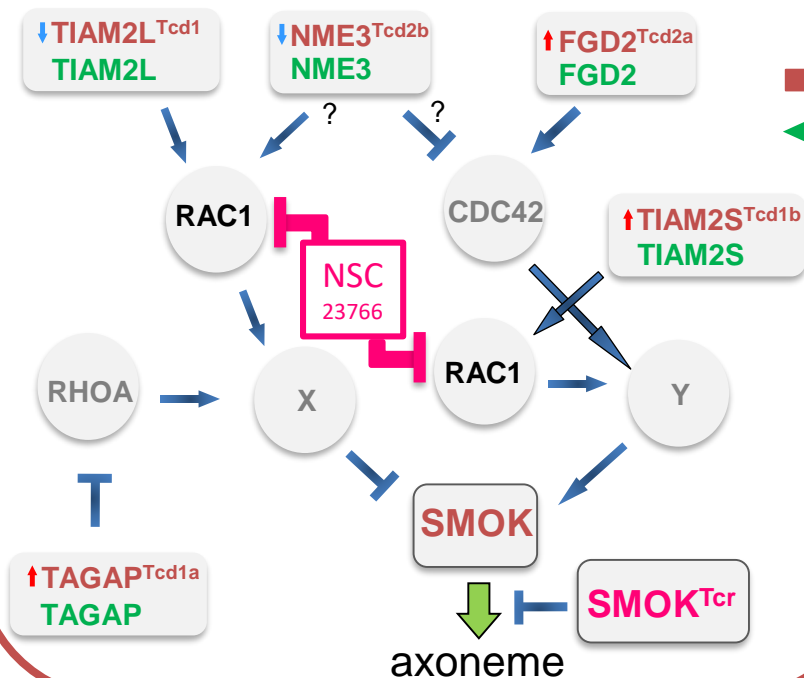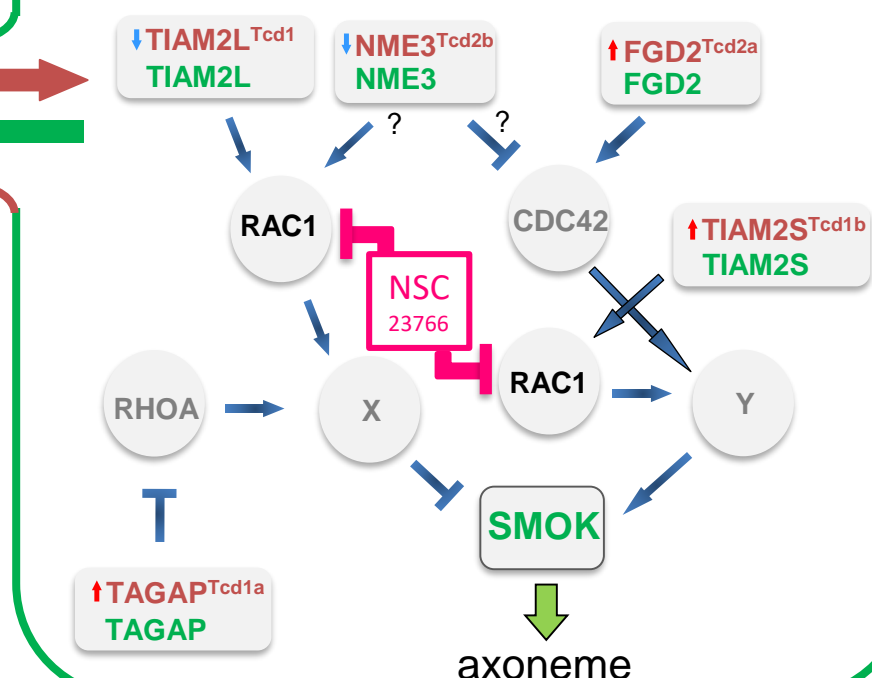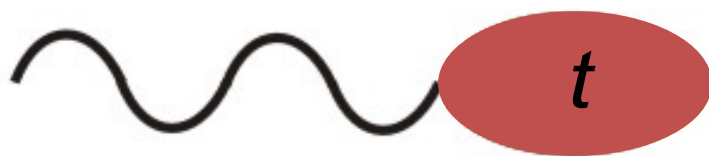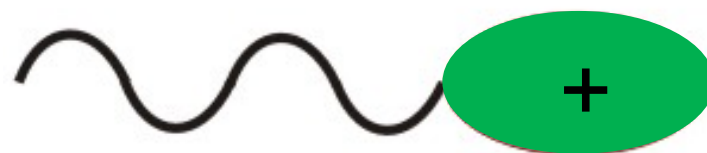

Supplement: S6 Fig — Schematic drawing representing two haploid spermatids connected by a cytoplasmic bridge allowing the exchange of RNA and proteins (horizontal arrows), expressing the wild type (+; genes and gene products in green) or t-haplotype (t; genes and gene products in dark red) variant of chromosome 17. Distorter gene products from either genotype act in both cells on Rho GTPase pathways effecting activation (through factor Y) or inhibition (through factor X) of SMOK, which controls sperm progressive motility. Upregulation of the activating pathway and parallel downregulation of the inhibitory pathway impair progressive motility in +-sperm, while t-sperm is protected by dominant-negative SMOKTCR, which is retained in and thus exclusively rescues progressive motility of t-sperm. Lower panel: The RAC1 inhibitor NSC23766 attenuates the effect of enhanced RAC1 activity caused by TIAM2S in both cells and thereby rescues progressive motility in +-sperm (in a strictly dosage dependent manner). Thus, it adopts the role of SMOKTCR missing in +-sperm. SMOK activity in t-sperm and +-sperm are approximated by NSC23766 (5 μM) treatment, therefore t-sperm and +-sperm are equalized with respect to progressive motility. RAC1 in the repressive pathway might be low due to down-regulation of the t-allele of TIAM2L, and thus, inhibition by NSC23766 might not significantly reduce the activity of factor X, which is also controlled by TAGAP [16]. Arrows indicate activation, blocked bars inhibition. Red upward pointing arrows at distorter proteins indicate up-regulation, blue down-pointing arrows down-regulation of the t variant; the green down-pointing arrow to the axoneme symbolizes normal or rescued, the dark-red down-pointing arrow impaired progressive motility. Grey gene symbols, X and Y indicate postulated factors. (PDF) [file pgen.1009308.s010.pdf]

## Slide 1
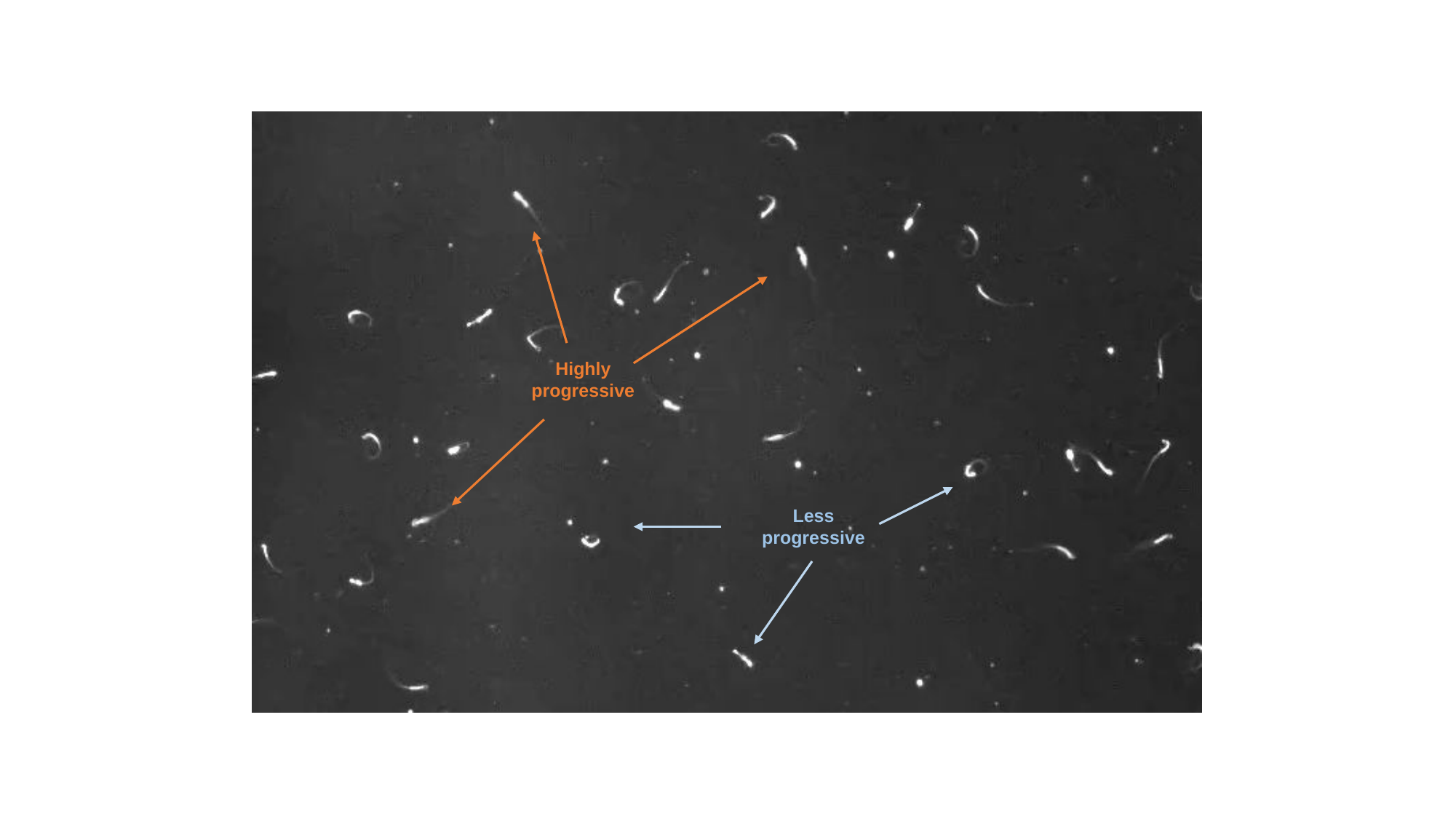

Highly progressive
Less progressive

Supplement: S10 Video — For training purposes tracks were omitted. Three highly progressive or less progressive sperm each are indicated. (PPTX) [file pgen.1009308.s020.pptx]
